# Rooting for function: community‐level fine‐root traits relate to many ecosystem functions

**DOI:** 10.1111/nph.70606

**Published:** 2025-10-03

**Authors:** Kathryn E. Barry, Justus Hennecke, Alexandra Weigelt, Joana Bergmann, Helge Bruelheide, Grégoire T. Freschet, Colleen M. Iversen, Thomas W. Kuyper, Daniel C. Laughlin, M. Luke McCormack, Catherine Roumet, Fons van der Plas, Jasper van Ruijven, Rachel Wijsmuller, Harald Auge, Nico Eisenhauer, Josephine Haase, Charles A. Nock, Yvonne Oelmann, Wolfgang Wilcke, Liesje Mommer

**Affiliations:** ^1^ Ecology and Biodiversity, Department of Biology Utrecht University Padualaan 8 3584 CH Utrecht the Netherlands; ^2^ Systematic Botany and Functional Biodiversity, Institute of Biology Leipzig University Johannisallee 21 04103 Leipzig Germany; ^3^ German Center for Integrative Biodiversity Research (iDiv) Halle‐Jena‐Leipzig Puschstr. 4 04103 Leipzig Germany; ^4^ Forest Ecology and Forest Management Group Wageningen University & Research PO Box 47 6700 AA Wageningen the Netherlands; ^5^ Leibniz Centre for Agricultural Landscape Research (ZALF) Eberswalder Straße 84 15374 Müncheberg Germany; ^6^ Institute of Biology/Geobotany and Botanical Garden Martin Luther University Halle‐Wittenberg Am Kirchtor 1 06108 Halle (Saale) Germany; ^7^ Theoretical and Experimental Ecology Station CNRS 2 route du CNRS 09200 09200 France; ^8^ Environmental Sciences Division Oak Ridge National Laboratory 1 Bethel Valley Rd #5200 Oak Ridge TN 37831 USA; ^9^ Soil Biology Group Wageningen University & Research PO Box 47 6700 AA Wageningen the Netherlands; ^10^ Department of Botany University of Wyoming 1000 E. University Ave Laramie WY 82071 USA; ^11^ Center for Tree Science The Morton Arboretum 4100 Illinois Route 53 Lisle IL 60532 USA; ^12^ CEFE, Univ Montpellier CNRS, EPHE, IRD 163 rue Auguste Broussonnet 34090 e Montpellier France; ^13^ Plant Ecology and Nature Conservation Wageningen University PO Box 47 6700 AA Wageningen the Netherlands; ^14^ Department of Community Ecology Helmholtz Centre for Environmental Research – UFZ Theodor‐Lieser‐Str. 4 06120 Halle Germany; ^15^ Experimental Interaction Ecology, Institute of Biology Leipzig University Puschstr. 4 04103 Leipzig Germany; ^16^ Department of Aquatic Ecology Eawag – Swiss Federal Institute of Aquatic Science and Technology Überlandstrasse 133 8600 Dübendorf Switzerland; ^17^ Department of Renewable Resources, Faculty of Agriculture, Life and Environmental Sciences University of Alberta 442 Earth Sciences Building Edmonton AB Canada T6G 2E3; ^18^ Geoecology, Department of Geosciences University of Tübingen Rümelinstr. 19‐23 72070 Tübingen Germany; ^19^ Institute of Geography and Geoecology Karlsruhe Institute of Technology (KIT) Reinhard‐Baumeister‐Platz 1 76131 Karlsruhe Germany

**Keywords:** biodiversity, ecosystem functions, fine roots, functional diversity, root economics space, root traits, trait‐functioning relationships

## Abstract

- Humans are driving biodiversity change, which also alters community functional traits. However, how changes in the functional traits of the community alter ecosystem functions—especially belowground—remains an important gap in our understanding of the consequences of biodiversity change.
- We test hypotheses for how the root traits of the root economics space (composed of the collaboration and conservation gradients) are associated with proxies for ecosystem functioning across grassland and forest ecosystems in both observational and experimental datasets from 810 plant communities. First, we assessed whether community‐weighted means of the root economics space traits adhered to the same trade‐offs as species‐level root traits. Then, we examined the relationships between community‐weighted mean root traits and aboveground biomass production, root standing biomass, soil fauna biomass, soil microbial biomass, decomposition of standard and plot‐specific material, ammonification, nitrification, phosphatase activity, and drought resistance.
- We found evidence for a community collaboration gradient but not for a community conservation gradient. Yet, links between community root traits and ecosystem functions were more common than we expected, especially for aboveground biomass, microbial biomass, and decomposition.
- These findings suggest that changes in species composition, which alter root trait means, will in turn affect critical ecosystem functions.

## Introduction

Global change is driving biodiversity change at unprecedented rates (Pörtner *et al*., 2021; Lee *et al*., 2023). This biodiversity change is composed not only of species loss at the global scale but also of changes in local‐scale species composition (Blowes *et al*., 2019). Plant diversity, one crucial piece of this changing diversity, supports many ecosystem properties, pools, and processes (hereafter, ecosystem functions, see Box 1) that are proxies for ecosystem functioning, from biomass production to carbon storage (Tilman *et al*., 2014; Isbell *et al*., 2015; Vogel *et al*., 2019; Miedema Brown & Anand, 2022). Changes in plant diversity are therefore likely to impact ecosystem functions (Tilman, 1999; Brauman *et al*., 2020).

Box 1 **Ecosystem functions in our dataset**

**Our definition of an ecosystem function** – Ecosystem properties, pools and processes, that are potentially influenced by plant communities and can be measured at or over a specific point in time in a plant community. These properties and processes are proxies for one or more facets of ecosystem functions. We acknowledge that the terminology of ecosystem functions has been used inconsistently in the literature (e.g. De Groot *et al*., 2002; Hoffland *et al*., 2020; Garland *et al*., 2021) and that the individual measures used in our study are not equally closely related to the functioning of the ecosystem. A detailed table of the original ecosystem function measures is presented in Supporting Information Table S2.

**Aboveground biomass production** – A proxy for vegetation net primary production, quantified as aboveground biomass production and measured as basal area increment over a given timeframe in forests and as the annually produced biomass in harvested clip plots in grasslands, standardized by site.

**Root standing biomass** – A proxy for root activity and influence in soil, includes quantification of root standing biomass in soil pits or in soil cores at different depths, at different times of the year, standardized by site.

**Soil fauna biomass** – A proxy for the biomass production of higher trophic level hetero/saprotrophs and predators of saprotrophic fauna, here the biomass of earthworms, standardized by site.

**Soil microbial biomass** – A proxy for soil microbial activity at a given site, includes measurements of microbial biomass quantified via phospholipid fatty acids and soil respiration, standardized for a given site. (Note that these measurements do not equally cover bacteria and fungi, as well as specific fungal guilds, such as mycorrhizal fungi).

**Decomposition – standard** – A proxy for the potential rate that material can be decomposed by the resident microbial community in the soil at a given site, includes the rate at which the microbial community at a given plot decomposed a standard material ranging from wood to tea bags, standardized by site.

**Decomposition – specific** – A proxy for carbon cycling rate at a given site, and may include the decomposition rate of leaf or root material from a given plot and site, allowed to decompose in the soil at that site taking potential effects of home‐field advantage into account, standardized by site.

**Ammonification** – A proxy for ecosystem nitrogen cycling, measured as net ammonification (sometimes also termed net nitrogen mineralization) or gross ammonification, in the laboratory or as buried soil cores (partly with root exclusion) in the field, standardized by site.

**Nitrification** – A proxy for ecosystem nitrogen cycling (or the abundance of nitrifying bacteria), measured as net nitrification, gross nitrification, or potential nitrification, in the laboratory or as buried soil cores in the field, standardized by site.

**Soil phosphatase activity** – A proxy for ecosystem phosphorus cycling, measured by the transformation of organic phosphorus compounds to orthophosphate, from sieved or unsieved soil (i.e. without live roots), standardized by site.

**Drought resistance of the plant community** – A proxy for stress resistance of the plant community, includes differences in delta ^13^C between wet and dry years, and calculations of the average ecosystem productivity in non‐drought years divided by the absolute value of the difference between ecosystem productivity during a drought event and the average ecosystem productivity in non‐drought years, standardized by site.

One key way in which changes in plant community diversity may manifest is through changes to the relative proportion of functional traits represented in the community (Díaz & Cabido, 2001). These shifts in functional community composition may occur when biodiversity change is driven non‐randomly (Lepš, 2004; Wardle *et al*., 2011). For example, increased N deposition leads to increased N availability, favoring species with traits typically associated with fast growth rates (Ellenberg, 1985; Endara & Coley, 2011).

Changes in the functional composition of plant communities will likely have direct and indirect effects on ecosystem functions (Chapin III *et al*., 2000). Yet, studies that aimed to understand ecosystem functions from a trait perspective have yielded mixed results. For example, van der Plas *et al*. (2020) found only weak relationships between traits and functioning across 41 plant functional traits and 42 functions collected over 15 years in a biodiversity experiment. However, studies that focus on specific trait–functioning relationships linked to well‐defined mechanisms better explain ecosystem functions (reviewed in Streit & Bellwood, 2023). For example, several studies show that specific leaf area and leaf nitrogen content can explain functions like aboveground primary productivity (Reich *et al*., 2012), turnover of soil organic carbon (Henneron *et al*., 2020a), and soil nitrogen cycling (Laughlin, 2011; Henneron *et al*., 2020b).

Many trait–functioning studies, however, tend to be constrained by three factors. First, the majority of studies on global trait patterns focus on data at the species level rather than at the community level (Díaz *et al*., 2016; Bergmann *et al*., 2020; Weigelt *et al*., 2021). However, this focus on species‐level data overestimates the importance of traits of rare species. Within a community, variation in abiotic and biotic conditions largely determines the relative abundance of different species in both space and time, ultimately determining how relevant a species is for ecosystem functions in a given environment (Díaz *et al*., 2007). Second, many papers focus only on data from biodiversity experiments which manipulate the species pool and deliberately minimize environmental variation at the local level (Schmid & Hector, 2004; Vogel *et al*., 2019; Jochum *et al*., 2020). Limiting environmental variation may also limit the covariation between traits and ecosystem functions because abiotic heterogeneity is a major driver of both community‐trait and ecosystem‐function variation (Laughlin *et al*., 2021). Therefore, the extent to which traits and functions can be related in experimental systems may be limited. Third, the majority of research on the links between functional traits and ecosystem functions focuses on aboveground traits (reviewed by Miedema Brown & Anand, 2022). Yet, many important components of ecosystem functions, including aspects of carbon, nitrogen, and water cycling, occur predominantly belowground and are dependent on plant roots and the soil microbial community (Keller *et al*., 2021; Freschet *et al*., 2021b). Further, recent evidence suggests that fine‐root traits may be better predictors of aboveground carbon storage and woody biomass productivity than leaf traits (Da *et al*., 2023). The focus on aboveground traits may bias our understanding of the general link between traits and ecosystem functions, particularly in ecosystems where the majority of plant biomass is located belowground and where aboveground processes are not a good proxy of belowground processes (Poorter *et al*., 2012). Thus, a better integration of root traits into trait–functioning relationships has the potential to significantly advance our understanding of ecosystem functions.

Recent progress in both our theoretical understanding of fine‐root traits (Bergmann *et al*., 2020; Weigelt *et al*., 2021; Freschet *et al*., 2021b) and practical access to root trait data (Iversen *et al*., 2017; Guerrero‐Ramírez *et al*., 2021; Freschet *et al*., 2021a) allows us to better assess relationships among root traits and ecosystem functions. In particular, the recent development of the root economics space (RES, Bergmann *et al*., 2020; Matthus *et al*., 2025) enables us to develop general hypotheses for how gradients in root trait space may be associated with ecosystem functions. Unlike the leaf economics spectrum, the RES has two orthogonal axes – the fungal collaboration gradient and the conservation gradient. The fungal collaboration gradient (hereafter the ‘collaboration’ gradient) ranges from species that invest in building thin but long roots with a high specific root length (SRL) on one side of the gradient and plants that invest in large diameter (MRD) roots on the other side of the gradient. The high SRL species are more likely to acquire resources themselves, while the larger‐diameter species are more likely to rely on mycorrhizal colonization (do‐it‐yourself vs outsourcing strategies). The conservation gradient is functionally similar to the traditional leaf economic spectrum (Wright *et al*., 2004; Weigelt *et al*., 2021). The conservation gradient ranges from plant species that invest in high root tissue density (RTD) on one side of the gradient to plant species that invest in a high root N content (RNC) on the other side of the gradient (Bergmann *et al*., 2020). High RTD species tend to invest in longer root lifespans, while high RNC species tend to have a higher root metabolism, growth rate, and turnover (Reich *et al*., 2008; Hou *et al*., 2024). This conceptual understanding of which fine‐root traits are predictors for plant functions and how they relate to each other allows us to construct mechanistic hypotheses for how these gradients relate to specific ecosystem functions.

For ecosystem functions, however, species‐level patterns may be less important than local community‐level patterns that depend on community composition and environmental conditions. Despite the extensive use of the conceptual RES in recent literature, it is unclear whether the two‐dimensional species‐level RES remains consistent at the community level (i.e. when weighting species traits by their relative abundances). Whereas species‐level trait patterns largely arise from interspecific eco‐evolutionary trade‐offs, community‐level trait patterns are mainly the result of community assembly processes (Anderegg, 2023). For the leaf economics spectrum, the pattern is independent of ecological scale; that is, it is both a species‐level and a community‐level pattern (e.g. Anderegg *et al*., 2018). Belowground, however, the RES has so far been most commonly assessed at the species level (Matthus *et al*., 2025). Some recent studies have partially confirmed the two root‐trait gradients for community‐level trait data (Da *et al*., 2023; Ma *et al*., 2024; Hennecke *et al*., 2025). Other studies, however, could not clearly demonstrate the conservation gradient at the community level (Prieto *et al*., 2015; Erktan *et al*., 2018; Lachaise *et al*., 2022). Alternatively, literature examining the individual traits that comprise the RES gradients provides initial support for strong trait–functioning relationships, suggesting that the community‐level RES may be relevant for ecosystem functioning. For example, recent evidence suggests that aboveground productivity in woody species is significantly higher in tree communities with ‘fast’ root traits (high RNC, Da *et al*., 2023).

Here, we examined the emergence of a community‐level RES and tested 40 individual hypotheses for community‐trait–function relationships (Table 1). The specific hypotheses were compiled based on the literature surrounding how the core traits of the RES (SRL, D, RTD, and RNC) relate to ten ecosystem functions (broadly related to carbon cycling and productivity, nutrient cycling, and stress resistance; see Table 1, also for references). We hypothesized that traits of the collaboration gradient would be associated with only three ecosystem functions (soil microbial biomass, specific decomposition, and nitrification; Box 1). Alternatively, we hypothesized that conservation gradient traits relate to eight ecosystem functions (aboveground biomass, soil fauna biomass, soil microbial biomass, decomposition of standard and specific material, ammonification, nitrification, and plant community drought resistance; Box 1). Overall, based on the literature, we were able to develop a larger number of specific hypotheses for the conservation axis than for the collaboration axis trait–functioning relationships; we therefore also expected that collaboration traits would explain fewer functions than conservation traits.

**Table 1:** Hypotheses and results with interpretations.

Trait–function hypotheses, rationale and results					
	Trait	Rationale	Hypothesis	Actual relationship	Explanation of actual relationship
Aboveground biomass production					
Collaboration	SRL	Small MRD and hence high SRL have, on theoretical grounds, been associated both with fertile soils (where plants are more frequently non‐mycorrhizal) and with extremely infertile soils (where fertility is so low that non‐mycorrhizal strategies) or alternative mycorrhizal strategies (ericoid mycorrhiza, ectomycorrhiza) are competitively superior. We therefore expect relationships of plant productivity and mycorrhizal colonization to be context‐dependent (Eissenstat, 1992) and might not allow for general patterns. We do not expect strong relationships.	nh	(−)	More productive environments potentially have higher soil N, which has a negative effect on SRL (in trees, Ostonen *et al*., 2007) while (potentially productive) species in grasslands with lower N also show reduced SRL (Craine *et al*., 2002). High N soils may similarly have a positive effect on MRD (Gao *et al*., 2023).
	MRD		nh	(+)	
Conservation	RTD	We expect a negative relationship because roots with a higher RTD allocate more carbon per unit root belowground (Bergmann *et al*., 2020), which is then not available for growth aboveground (Poorter *et al*., 2012). Additionally, high RTD is often associated with high leaf mass area (low specific leaf area) and an overall more conservative growth strategy (Kramer‐Walter *et al*., 2016; Weigelt *et al*., 2021).	(−)	(−)	As hypothesized.
	RNC	We expect a positive relationship because RNC is positively correlated to leaf nitrogen content which is positively correlated with photosynthetic rate (Weigelt *et al*., 2021). Although above‐ vs belowground biomass allocation may vary, roots with higher RNC are fast growing species (Weigelt *et al*., 2021), and may therefore be more productive.	(+)	(+)	While the direction of effect is as hypothesized, the effect is weaker than expected. The high variability in trait–function relationship indicates that higher RNC is not always beneficial to productivity. This is in line with Augusto *et al*. (2025), who have recently shown that a conservative aboveground strategy is associated with higher growth rates in unfavorable conditions, and acquisitive strategies are only more productive under certain environmental conditions.
Root standing biomass					
Collaboration	SRL	Root standing biomass is a product of root productivity and root longevity. These properties may be negatively correlated – thicker roots have a longer lifespan than thinner roots but may contribute less to productivity. Thick roots may also be proportionally more N‐rich, further counteracting increased lifespan. As we do not know which of these mechanisms has the strongest effects, it is difficult to hypothesize how collaboration‐related root traits will affect root standing biomass.	nh	ne	As no relationship was observed, we suggest that the trade‐off between root longevity (higher with high MRD) and root productivity (lower with high MRD) was balanced in this data set but could vary systematically within communities.
	MRD		nh	ne	
Conservation	RTD	Three mechanisms may interact in opposite directions. First, a positive relationship between RNC and RSB is expected when roots with a higher RNC represent fast growing species in fertile ecosystems, and produce high RSB. Second, this relationship may be weakened as the relative investment in roots decreases in fertile ecosystems, although overall root biomass may still be increased due to extra demand for nutrients (Poorter *et al*., 2012). Third, this relationship can be further weakened because root longevity is expected to be shorter in fertile systems (McCormack & Guo, 2014). The relative weight of each mechanism is impossible to determine, hence no hypothesis.	nh	ne	This result may indicate that multiple counteracting mechanisms ultimately sum to a net zero effect on functioning.
	RNC		nh	ne	
Soil fauna biomass					
Collaboration	SRL	Initial searches identified insufficient literature to develop hypotheses for these root traits.	nh	ne	Previous studies found that experimental addition of earthworms (the main soil fauna proxy in our dataset) can decrease MRD/increase root length (Agapit *et al*., 2018; Junaidi *et al*., 2018).
	MRD		nh	ne	
Conservation	RTD	In general, soil fauna is less active or limited in systems that are poor in nutrients resulting from low litter decomposability (i.e. high RTD) (Wardle *et al*., 2004).	(−)	ne	Despite expected low palatability of high RTD litter, no relationship was found. This could be because high RTD selects for fungi over bacteria and might therefore also benefit fungivorous organisms like earthworms.
	RNC	We expect a strongly positive relationship as both root and leaf litter (due to the coupling of RNC with leaf NC (Weigelt *et al*., 2021)), provide high quality input for soil fauna.		(+)	As hypothesized.
Soil microbial biomass					
Collaboration	SRL	Although previous studies partly failed to identify strong links between root traits and microbial biomass (De Long *et al*., 2019), we expect a positive relationship. Species with high SRL should produce higher root length densities (RLD), compared to species with low SRL, and RLD is positively correlated to soil microbial biomass (Lange *et al*., 2015). The increased root length and root surface area with high SRL could provide increased exudation surfaces to further stimulate microbial biomass (Guyonnet *et al*., 2018; Gao *et al*., 2024)	(+)	ne	Effects of SRL on microbial biomass seem to be weaker than that of other traits. If mycorrhizal colonization decreases with SRL, mycorrhizal fungal biomass in the soil likely also decreases. Furthermore, as aboveground productivity decreases with SRL, there is potentially less litter available to the microbial community. Overall, these multiple mechanisms seem to sum up to no net effect.
	MRD	While MRD may positively correlate with microbial activity in rhizosphere soil (Borden *et al*., 2021), we expect a negative relationship in bulk soil as MRD is negatively related with RLD and should therefore decrease exudation due to smaller surface area (see above).	(−)	(+)	Recent studies found increased rhizodeposition (Folacher *et al*., 2024) and exudation (Williams *et al*., 2022) with high MRD. Further, increased mycorrhizal colonization could increase soil microbial biomass (Barceló *et al*., 2020). Labile C and N stored in the larger root cortex might be beneficial to the soil microbial community. Furthermore, as aboveground productivity increases with MRD, there is potentially more litter available to the microbial community.
Conservation	RTD	Litter from higher RTD communities correlates with increased lignin content which results in low carbon use efficiency and reduced biomass accumulation (Sinsabaugh *et al*., 2013). Lower exudation rates should further result in decreased microbial biomass (Guyonnet *et al*., 2018).	(−)	(−)	As hypothesized.
	RNC	Microbial biomass is stimulated by litter with high RNC (Wardle *et al*., 2004). Productive communities with high RNC can indirectly stimulate microbial biomass via increased leaf area and soil shading resulting in increased soil moisture and improved habitat for microbes (Lange *et al*., 2014). High RNC was shown to increase exudation (Sun *et al*., 2021). Soil microbial biomass N limitation is reduced; higher N leads to higher C use efficiency of microbes (Sinsabaugh *et al*., 2013).	(+)	ne	Although weakly positive, RNC surprisingly did not have a relevant effect on soil microbial biomass. This could mean that N limitation is not as relevant as previously thought; or this could be because the effect of N is mediated by interactions between the dominant C source type and soil pH, so only having an indirect effect on (at least) decomposer communities (Hall *et al*., 2020). Productive communities with increased leaf area might also have negative effects on SMB due to increased evapotranspiration and soil drying in some ecosystems (Serna‐Chavez *et al*., 2013; Zeng *et al*., 2018).
Decomposition – Standard material					
Collaboration	SRL	It is difficult to formulate a hypothesis due to the strong interactions between various ‘standard’ materials and soil properties which might obscure the effects of plant traits.	nh	(+)	The rhizosphere effect on SOM decomposition may relate negatively to MRD (and hence positively to SRL), increasing soil priming and decomposition (Han *et al*., 2020). The negative relationship with MRD could be due to poorly adapted microbiota as MRD is positively related to specific decomposition (see below).
	MRD		nh	(−)	
Conservation	RTD	Decomposition scales with N availability where N is limiting and when the ‘standard’ material has a high C : N ratio but low lignin (Berg, 2000; Sun *et al*., 2018); high community RTD is related to low productivity, ‘slow’ systems with reduced rate of decomposition.	(−)	(+)	While roots with high RTD are less decomposable, they might select for microbial communities adapted to low litter quality, as found in the standard material used in many studies (‘home‐field advantage’ for standard material in communities with high RTD compared to plots with high RNC).
	RNC	Increased RNC relates to more productive systems with active microbial communities, therefore likely increased decomposition. Decomposition is less likely to be N‐limited in systems with high RNC species (Taylor *et al*., 1991).	(+)	(−)	Even though decomposition is not N‐limited, the specific assemblage of microbiota may not be well adapted to the supplied material (which is often low in RNC).
Decomposition – Plot‐specific material					
Collaboration	SRL	High SRL root systems have more surface area available for attack and decomposition; however, thin, fine roots will have proportionally more stelar tissue (Bergmann *et al*., 2020) which is more lignified and less decomposable than cortex (See *et al*., 2019; Xia *et al*., 2021). Overall, we hypothesize a weakly positive relationship.	(+)	ne	Effects of mycorrhizal fungi may be difficult to disentangle for this trait due to the promoting and retarding actions of mycorrhizal fungi on decomposition (Kuyper & Jansa, 2023). Previous studies suggested decomposability is more strongly related to root chemical traits rather than morphology (Birouste *et al*., 2012).
	MRD	Cortical thickness increases disproportionately in thicker roots creating more easily decomposable, potentially less dense tissue, which can promote decomposition (Kong *et al*., 2014, 2016; Jimoh *et al*., 2024).	(+)	ne	This relationship varies strongly across sites suggesting that it is context dependent which cannot be further resolved by our dataset.
Conservation	RTD	Roots with a higher RTD are in general more lignified, and thus more difficult to decompose (Silver & Miya, 2001). The strength of this relationship might be related to the variation in root decomposition rates across root orders (Goebel *et al*., 2011) and additional variation in root C types beyond lignin content (Sun *et al*., 2018).	(−)	ne	This relationship varies strongly across sites suggesting that it is context dependent which cannot be further resolved by our dataset.
	RNC	We expect a positive relationship because roots with high RNC are more easily decomposed due to lower C:N ratio (Silver & Miya, 2001; Sun *et al*., 2018; Jimoh *et al*., 2024).	(+)	(+)	This strongly positive relationship supports the argument that removal of N limitation and increased productivity in high RNC communities enable *well‐adapted* microbiota to increase specific decomposition, whereas with standard material, removing N‐limitation did not result in improved decomposition.
Ammonification					
Collaboration	SRL	Initial searches identified insufficient literature to develop hypotheses for these root traits.	nh	ne	Root – ammonification linkages may be indirect and, for example, can be mediated by root hydraulic properties and water availability (Cardon *et al*., 2013). They may also operate via soil organic matter (SOM) dynamics and mycorrhizal behavior (Phillips *et al*., 2013) and be subject to legacy effects governing the quality and quantity of SOM, especially when measurement methods remove active roots.
	MRD		nh	(+)	
Conservation	RTD		nh	ne	
	RNC	Lower C:N ratio of litter promotes higher N mineralization rates (in grasslands) (Lama *et al*., 2020; Man *et al*., 2020). Presence of legumes (usually with high RNC) increases ammonification via increased N availability (Lama *et al*., 2020).	(+)	(+)	As hypothesized.
Nitrification					
Collaboration	SRL	Competition between plants and nitrifying bacteria occurs in grasslands as root N uptake increases with increased SRL. More root uptake reduces remaining NH_4_ available for nitrification (Cantarel *et al*., 2015).	(−)	(−)	As SRL and MRD correlate with nitrification but not ammonification, increased oxygen availability in soil resulting from larger pores induced by thick roots might explain increased nitrification (Bollmann & Conrad, 1998; Bodner *et al*., 2014). Additionally, MRD is positively related to microbial biomass, potentially also stimulating the community of nitrifying bacteria.
	MRD	Initial searches found insufficient literature to develop hypotheses for these root traits.	nh	(+)	
Conservation	RTD	Initial searches found insufficient literature to develop hypotheses for these root traits.	nh	(−)	In observational studies RTD can express habitat quality, where high RTD indicates lower nutrient availability and less N overall available for nitrification, as well as low nitrifier abundance (Legay *et al*., 2014). Further, increased C:N ratio of litter (with high RTD) might slow down N cycling due to lower N availability (Lama *et al*., 2020).
	RNC	RNC as an expression of habitat quality suggests a positive relationship between RNC and nitrification, related to nutrient rich environments and exploitative plant strategies (Wright *et al*., 2004). Habitats with high RNC are likely to have a higher pH (Read, 1991) which is positively correlated with nitrification as many nitrifiers are not acid‐tolerant (Haynes, 1986). Further, decreased C : N ratio of litter (with high RNC) should increase N cycling due to higher N availability (Orwin *et al*., 2010; Laughlin, 2011; Lama *et al*., 2020).	(+)	ne	While ammonification and nitrification show similar trait–function relationships (with the same direction of effects) in our dataset, we have more data on nitrification from observational systems compared to ammonification. In these systems, differences in historical land‐use and hence SOM formation might obscure patterns of plant traits (Compton & Boone, 2000).
Soil phosphatase activity					
Collaboration	SRL	No hypothesis for these traits because most methodologies remove roots to measure soil organic matter‐related enzyme activity, rather than that directly related to plant roots. Furthermore, disentangling the various sources of phosphatase in the soil (roots, AMF, and saprotrophs) was not possible and so no rationale for the impact of root traits could be identified.	nh	(−)	Han *et al*. (2022) found that root phosphatase aligned with the collaboration gradient in forests – this finding may be unsupported here due to methodological challenges.
	MRD		nh	ne	
Conservation	RTD		nh	(−)	This result aligns with previous studies on root phosphatase activity that suggested lower phosphorus mobilization of species with high RTD and low RNC (Ushio *et al*., 2015; Guilbeault‐Mayers & Laliberté, 2024), potentially through decreased metabolic activity.
	RNC		nh	ns	
Drought resistance of plant communities					
Collaboration	SRL	Species with high SRL show high specific surface area to take up water. Yet, species with high SRL are often less colonized by mycorrhizal fungi, which provide drought resistance through multiple mechanisms (Ruiz‐Lozano *et al*., 2012). No clear hypothesis as these traits may both confer drought resistance through different mechanisms.	nh	ne	Although it has previously been found that plants increase MRD in response to drought (Zhou *et al*., 2018) and mycorrhizal associations generally enhance drought resistance and tolerance (Ruiz‐Lozano *et al*., 2012), in fact multiple strategies may confer drought resistance to plants in different environments, resulting in unclear trait trade‐offs and no strong relationship (Lozano *et al*., 2020; Laughlin *et al*., 2021). This is supported by previous studies which identified a variety of adaptive strategies occupying a large root phenotypic morphospace (Valverde‐Barrantes & Blackwood, 2016). As an example, while higher RTD protects roots from dehydration damage, the development of cortical aerenchyma and low RTD is also an effective drought resistance strategy (Klein *et al*., 2020). Other traits related to hydraulic capacity of plants, such as rooting depth or hydraulic conductivity, might be more relevant for drought tolerance than traits of the RES.
	MRD		nh	ne	
Conservation	RTD	Higher RTD is associated with increased tissue resistance to cavitation and shrinkage under drought stress (Wahl & Ryser, 2000; Pittermann *et al*., 2006). Grasses with a low cortex: stele ratio show improved drought resistance due to reduced metabolic costs of root elongation in drought conditions since stelar tissue is less metabolically active and has lower water demands than cortical tissue (Yamauchi *et al*., 2021).	(+)	ne	
	RNC	Initial searches found insufficient literature.	nh	ne	

MRD, mean root diameter; ne, no effect; nh, no hypothesis; RTD, root tissue density; RNC, root nitrogen content; SRL, specific root length.

Where a hypothesis was made for one side of an axis, the opposite direction of effect is not automatically hypothesized to the other side of the axis for two reasons: (1) the mechanism may be unrelated to the axis, and only to the specific trait, (2) not all trait–functioning relationships are bidirectional (Laughlin *et al*., 2021); some relationships may only go one way (i.e. the trait supports function in only one extreme and has no effect at the other).

## Materials and Methods

### Literature review and hypothesis formulation

Before formalizing our analysis, we compiled a list of proxies for ecosystem functions commonly used in analyses of ecosystem functioning. These proxies cover major ecosystem functions such as plant productivity, carbon sequestration, nitrogen and phosphorus cycling, and soil microbial activity, which will hereafter be referred to as ‘ecosystem functions’ (Box 1). We developed *a priori* hypotheses for how these functions related to the four traits of the RES: specific root length (SRL), mean root diameter (MRD), root tissue density (RTD), and root nitrogen content (RNC). From this list, we selected 10 ecosystem functions that were commonly studied across systems. The final list of 10 functions included aboveground biomass production, root standing biomass, soil fauna biomass, soil microbial biomass, decomposition of standard material (e.g. filter paper), decomposition of litter composed of the species in the plot, ammonification rate, nitrification rate, soil phosphatase activity, and drought resistance (Box 1). For these functions, a preliminary literature search indicated a high likelihood of sufficient data across systems ranging from grasslands to forests.

Based on our preliminary literature review, we formalized our hypotheses for the four resource economics space traits in relation to each of the 10 functions. While we selected these functions because the literature suggested there would be sufficient publicly available data, there was not necessarily sufficient evidence in the literature to make hypotheses for the direction and/or strength of relationships for all 40 trait–function combinations. Because these hypotheses were based on existing literature that in some cases preceded the root economic space, where a hypothesis was made for one trait of an axis, we did not automatically apply the opposite to the other trait of the axis. For example, if we hypothesized that a function was positively correlated with RTD, we did not automatically assume it would be negatively correlated with RNC just because the traits themselves are often negatively correlated. We therefore distinguished between ecological gradients like the collaboration and conservation gradients with their antagonistic functional strategies at both ends, and the traits that serve as proxies for these gradients. The gradients present the more integral ecological strategies of a species/community, while the single traits represent individual hypotheses for trait–functioning relationships (Table 1).

### Ecosystem function data selection

Once we selected the functions of interest, our goal was to find datasets where as many of the 10 selected ecosystem functions were collected at the same site as possible to maximize comparability among functions (see Supporting Information Table S1 for all data sources and Table S2 for the full list of measures for each project). To minimize potential system bias by having unequal datasets across biomes, we deliberately selected experimental and observational studies in both forest and grassland ecosystems. The resulting set of studies included measurements of multiple functions as well as the assessment of species composition but was largely located in the temperate zone with some individual sites in boreal forests, tropical seasonal forests, or savannas. Studies that had multiple locations were coded as the same ‘project’ (e.g. Biodiversity Exploratories, and NEON), and individual locations were coded as different ‘sites’ within the project (e.g. the three ‘sites’ of the Biodiversity Exploratories across Germany, Fischer *et al*., 2010). When a project only comprised a single site (e.g. Jena Experiment), the project and site were labeled identically. Individual spatial units within a site where functions and species composition were measured were coded as ‘plots’.

### Ecosystem function standardization

To help us focus on root trait effects on ecosystem functions within sites, we accounted for macro‐environmental differences by centering and scaling all function data to unit variance within each ecosystem and project site. This standardization removed the variation in ecosystem functions among sites, for example, due to underlying differences in the abiotic conditions of each individual site. By standardizing in this way, we removed large‐scale differences in ecosystem functions that may underlie large observational gradients and are therefore not easily assigned to changes in the plant community or are more likely associated with climate and edaphic conditions. This standardization also allowed us to compare functions with multiple measurement methods (e.g. aboveground biomass production measured as an increase in basal area in trees vs by vegetation clipping in a grassland) that would not be comparable otherwise.

### Trait data compilation, standardization, and plot selection

To maximize the potential match between traits and ecosystem functions, we used plot‐level aboveground species composition data from the year in which the highest number of functions were measured at a site. We standardized species names using the Taxonomic Name Resolution Service, accessed through the ‘TNRS’ R package (Boyle *et al*., 2013). Plant species were matched at the species level with the extended root trait database of Weigelt *et al*. (2021), based on GRooT (Guerrero‐Ramírez *et al*., 2021). From this database, we collected data for SRL, MRD, RTD, and RNC. Once the full dataset was assembled, we removed plots from our analysis where < 80% of the plant community in a plot (see details below on species abundance data) had data for all four RES traits (Pakeman & Quested, 2007). The complete list of plot numbers included per site and function is found in Tables S3 and S4. All data manipulation and analyses were done in R v.4.3.2 (R Core Team, 2023).

### Data analysis

We calculated community‐weighted mean (CWM) traits from the species‐level traits, weighted by a metric of aboveground species abundance. Depending on data availability across studies, the relative abundance of a species was calculated using either aboveground biomass, aboveground cover, or species‐specific tree diameter at breast height (Table S2). These different measurements reflect the conventions that are most practical, and therefore most commonly used, in different systems. We then standardized these community‐weighted means at the level of the study and site to reduce the likelihood that using different measures for the community composition would alter our results. We examined the presence of a species‐level (based on unstandardized species traits) and community‐level (based on CWM traits standardized at the site level) RES in our data with a principal component analysis (PCA) based on a correlation matrix using the ‘princomp’ command in the R base ‘stats’ package. For the species‐level PCA, we centered and scaled trait data across the whole dataset. For the community‐level PCA, we centered and scaled trait data to unit variance within an ecosystem and site to enable better comparison across traits and ecosystems. A community‐level PCA without the 80% cutoff for trait data availability can be found in Fig. S1. For comparability, we did not use phylogenetic correction for the species‐level PCA, as this would not be possible at the community level (Lachaise *et al*., 2022; Da *et al*., 2023; Hennecke *et al*., 2025).

To test our individual hypotheses for trait–functioning relationships while avoiding multicollinearity, we used separate models for each trait–function combination, resulting in a total of 40 separate models. We fitted a series of linear Bayesian hierarchical models using the ‘brms’ package (Bürkner, 2017). For each trait–function combination, models included a fixed effect of the trait and a hierarchical term for site‐specific variation $\left(\text{ecosystem function}\sim \text{trait}+\left(0+\text{trait}\;|\;\text{site}\right)\right)$. Due to the z‐transformation of the function and trait data at the site level, the intercepts were all equal to or close to zero, and we therefore did not include random intercepts. We fitted the model using the Student‐t likelihood distribution to accommodate potential outliers and with a weakly informative normal distribution prior for both the fixed effects and the SD of the hierarchical effect. Posterior distributions were sampled using four chains of 4000 iterations each (1000 warm‐up), with adapt_delta=0.99. Model convergence was verified via Rhat values and posterior predictive checks (Fig. S2). All Rhat values were < 1.01 with sufficiently large bulk and tail effective sample sizes (Vehtari *et al*., 2021; Table 2). To evaluate the effect size and direction, we extracted the posterior mean of the population‐level slope, along with 89% credible intervals. We then used the posterior probability of direction (PD), defined as the proportion of posterior samples on the same side of zero, as an evidence metric for directional effects (Makowski *et al*., 2019). We categorized evidence strength as *moderate* (PD > 0.9), *strong* (PD > 0.95), or *very strong* (PD > 0.975). Posterior distributions are shown in Fig. S3. Predictions were generated for both global and site‐specific models over the observed trait range.

**Table 2:** Posterior mean estimates for the effect of the root trait on the ecosystem function, convergence diagnostics, and evidence strength.

Ecosystem function	Trait	Estimate	SD	89% CI	*R* ^2^	n_Obs_	n_Group_	Rhat	ESS (bulk)	ESS (tail)	PD	Evidence level
Aboveground biomass production	SRL	−0.316	0.075	[−0.429–0.193]	0.166	718	18	1.001	4291	5528	1.000	Very strong
Aboveground biomass production	MRD	0.245	0.093	[0.100 0.391]	0.144	718	18	1.002	2448	4550	0.994	Very strong
Aboveground biomass production	RTD	−0.353	0.095	[−0.506–0.204]	0.200	718	18	1.000	2606	4024	1.000	Very strong
Aboveground biomass production	RNC	0.166	0.107	[0.001 0.340]	0.122	718	18	1.001	2549	4249	0.946	Moderate
Root standing biomass	SRL	−0.009	0.056	[−0.096 0.080]	0.012	463	19	1.000	7257	6965	0.570	No effect
Root standing biomass	MRD	0.038	0.085	[−0.095 0.173]	0.054	463	19	1.001	4413	6088	0.682	No effect
Root standing biomass	RTD	0.054	0.069	[−0.057 0.157]	0.024	463	19	1.000	5385	4911	0.804	No effect
Root standing biomass	RNC	0.019	0.079	[−0.098 0.151]	0.038	463	19	1.001	3740	4818	0.582	No effect
Soil fauna biomass	SRL	0.118	0.107	[−0.046 0.285]	0.078	353	11	1.001	3082	4363	0.885	No effect
Soil fauna biomass	MRD	0.025	0.089	[−0.119 0.161]	0.037	353	11	1.001	4785	5477	0.625	No effect
Soil fauna biomass	RTD	0.072	0.068	[−0.037 0.176]	0.026	353	11	1.001	5573	4833	0.872	No effect
Soil fauna biomass	RNC	0.291	0.077	[0.170 0.411]	0.124	353	11	1.000	4755	5332	0.999	Very strong
Soil microbial biomass	SRL	−0.063	0.059	[−0.153 0.032]	0.022	524	21	1.000	8080	7641	0.862	No effect
Soil microbial biomass	MRD	0.104	0.072	[−0.012 0.216]	0.050	524	21	1.001	5945	7266	0.926	Moderate
Soil microbial biomass	RTD	−0.139	0.080	[−0.269–0.013]	0.072	524	21	1.000	6230	7749	0.960	Strong
Soil microbial biomass	RNC	0.059	0.053	[−0.024 0.142]	0.013	524	21	1.000	11 120	8928	0.875	No effect
Decomposition – standard material	SRL	0.107	0.045	[0.036 0.180]	0.017	587	16	1.000	15 027	8839	0.991	Very strong
Decomposition – standard material	MRD	−0.116	0.055	[−0.203–0.033]	0.024	587	16	1.001	7727	6181	0.984	Very strong
Decomposition – standard material	RTD	0.123	0.047	[0.050 0.197]	0.023	587	16	1.000	8684	7315	0.994	Very strong
Decomposition – standard material	RNC	−0.112	0.058	[−0.204–0.020]	0.031	587	16	1.001	6530	6997	0.973	Strong
Decomposition – plot‐specific material	SRL	0.046	0.078	[−0.069 0.171]	0.021	406	12	1.000	7055	5693	0.726	No effect
Decomposition – plot‐specific material	MRD	0.115	0.118	[−0.075 0.300]	0.102	406	12	1.001	3865	5499	0.846	No effect
Decomposition – plot‐specific material	RTD	−0.083	0.109	[−0.252 0.092]	0.075	406	12	1.000	3881	5388	0.797	No effect
Decomposition – plot‐specific material	RNC	0.292	0.092	[0.146 0.433]	0.132	406	12	1.000	4231	4803	0.995	Very strong
Ammonification	SRL	−0.071	0.097	[−0.216 0.081]	0.020	183	11	1.001	7161	5659	0.795	No effect
Ammonification	MRD	0.148	0.113	[−0.013 0.330]	0.037	183	11	1.000	5366	4671	0.932	Moderate
Ammonification	RTD	−0.117	0.115	[−0.287 0.065]	0.043	183	11	1.000	5905	5890	0.860	No effect
Ammonification	RNC	0.153	0.110	[−0.021 0.312]	0.047	183	11	1.000	6847	5258	0.928	Moderate
Nitrification	SRL	−0.089	0.070	[−0.200 0.020]	0.019	278	14	1.000	9890	7540	0.906	Moderate
Nitrification	MRD	0.161	0.092	[0.022 0.310]	0.049	278	14	1.001	5106	5167	0.967	Strong
Nitrification	RTD	−0.132	0.096	[−0.276 0.019]	0.047	278	14	1.000	6008	5382	0.925	Moderate
Nitrification	RNC	0.074	0.087	[−0.057 0.211]	0.027	278	14	1.001	7037	5684	0.820	No effect
Phosphatase activity	SRL	−0.110	0.077	[−0.231 0.010]	0.026	238	10	1.001	6032	5648	0.931	Moderate
Phosphatase activity	MRD	0.027	0.074	[−0.088 0.142]	0.015	238	10	1.000	7109	6080	0.654	No effect
Phosphatase activity	RTD	−0.189	0.066	[−0.295–0.087]	0.042	238	10	1.001	8881	7264	0.997	Very strong
Phosphatase activity	RNC	−0.040	0.086	[−0.173 0.093]	0.024	238	10	1.002	5372	5245	0.694	No effect
Drought resistance of the plant community	SRL	0.046	0.081	[−0.066 0.182]	0.014	398	10	1.000	3566	3395	0.734	No effect
Drought resistance of the plant community	MRD	−0.041	0.056	[−0.124 0.047]	0.008	398	10	1.000	6247	5044	0.801	No effect
Drought resistance of the plant community	RTD	−0.073	0.089	[−0.224 0.051]	0.020	398	10	1.001	3531	3831	0.823	No effect
Drought resistance of the plant community	RNC	−0.062	0.068	[−0.148 0.053]	0.013	398	10	1.001	4058	2957	0.860	No effect

CI, credible interval; CWM, community‐weighted mean; ESS, effective sample size; MRD, mean root diameter; PD, probability of direction; *R* ^2^, Bayesian *R* ^2^; Rhat, parameter of model convergence; RTD, root tissue density; RNC, root nitrogen content; SD, posterior SD; SRL, specific root length.

Traits and ecosystem functions were z‐transformed for each study site to account for differences in biotic (e.g. differences across ecosystems) and abiotic variation. Individual Bayesian hierarchical models for each combination of root traits and ecosystem function were fitted. Each model included a fixed effect of the trait and a random slope for site‐specific variation. The level of evidence was assigned based on PD ≤ 0.9 = no evidence of effect; 0.9 < PD < 0.95 = moderate evidence; 0.95 < PD < 0.975 = strong evidence; PD > 0.975 = very strong evidence.

## Results

### The RES at the species and the community level

**Fig. 1:**
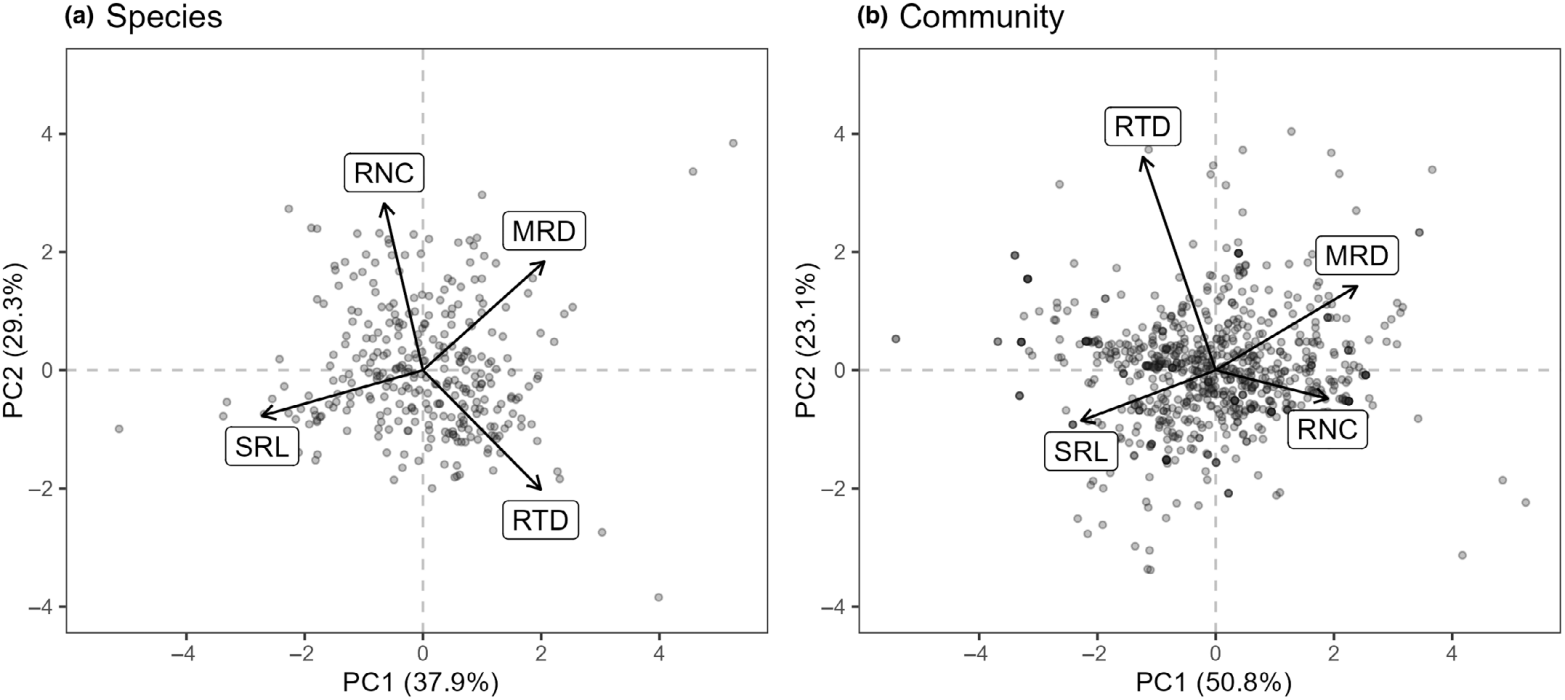
Community‐weighted mean trait values do not follow the same organization as at the species level. (a) Species‐level principal component analysis (PCA)– at the species level (317 species), we found that both principal components (PC 1 and PC 2) closely resembled the root economics space of Bergmann *et al*. (2020) with specific root length (SRL) and mean root diameter (MRD) representing the collaboration gradient, and root tissue density (RTD) and root nitrogen concentration (RNC) representing an orthogonal conservation gradient. (b) Community‐level PCA (810 plant communities). Note that all points are semi‐transparent to better visualize the overlap but appear darker when there are multiple overlapping points.

At the species level (317 species), we found that the coordination of SRL, MRD, RTD, and RNC was largely aligned with the RES *sensu* Bergmann *et al*. (2020) (Fig. 1a). SRL loaded more on PC1 (−0.680) than on PC2 (−0.192). MRD loaded more on PC1 (0.513) than PC2 (0.460), though by a smaller margin. RNC loaded more on PC2 (0.706) than on PC1 (−0.164), while RTD loaded similarly on PC1 (0.498) and PC2 (−0.504). PC1 accounted for 37.9% of the variance in our species data, while PC2 accounted for 29.3% of the variance for a cumulative 67.2% (PC3: 20.7%).

At the community level (810 communities), the RES differed considerably from the species level, with RNC_CWM_ loading together with MRD_CWM_ rather than RTD_CWM_ (Fig. 1b). SRL_CWM_ and MRD_CWM_ showed a similar pattern to the species level, loading more on PC1 (−0.569 and 0.598, respectively) than on PC2 (−0.214 and 0.356, respectively), and RTD_CWM_ loaded more on PC2 (0.902) than on PC1 (−0.308). However, RNC_CWM_ did not follow the species‐level organization but rather loaded more strongly on PC1 (0.474) than PC2 (−0.120). Further, RNC_CWM_ loaded even more strongly on PC3 (−0.827), though PC3 was not needed to account for sufficient variance in the model. At the community level, PC1 explained 50.8% of the variance in our data, while PC2 accounted for 23.1%, for a cumulative 73.9% of the variance.

### Root trait – Ecosystem function relationships

**Fig. 2:**
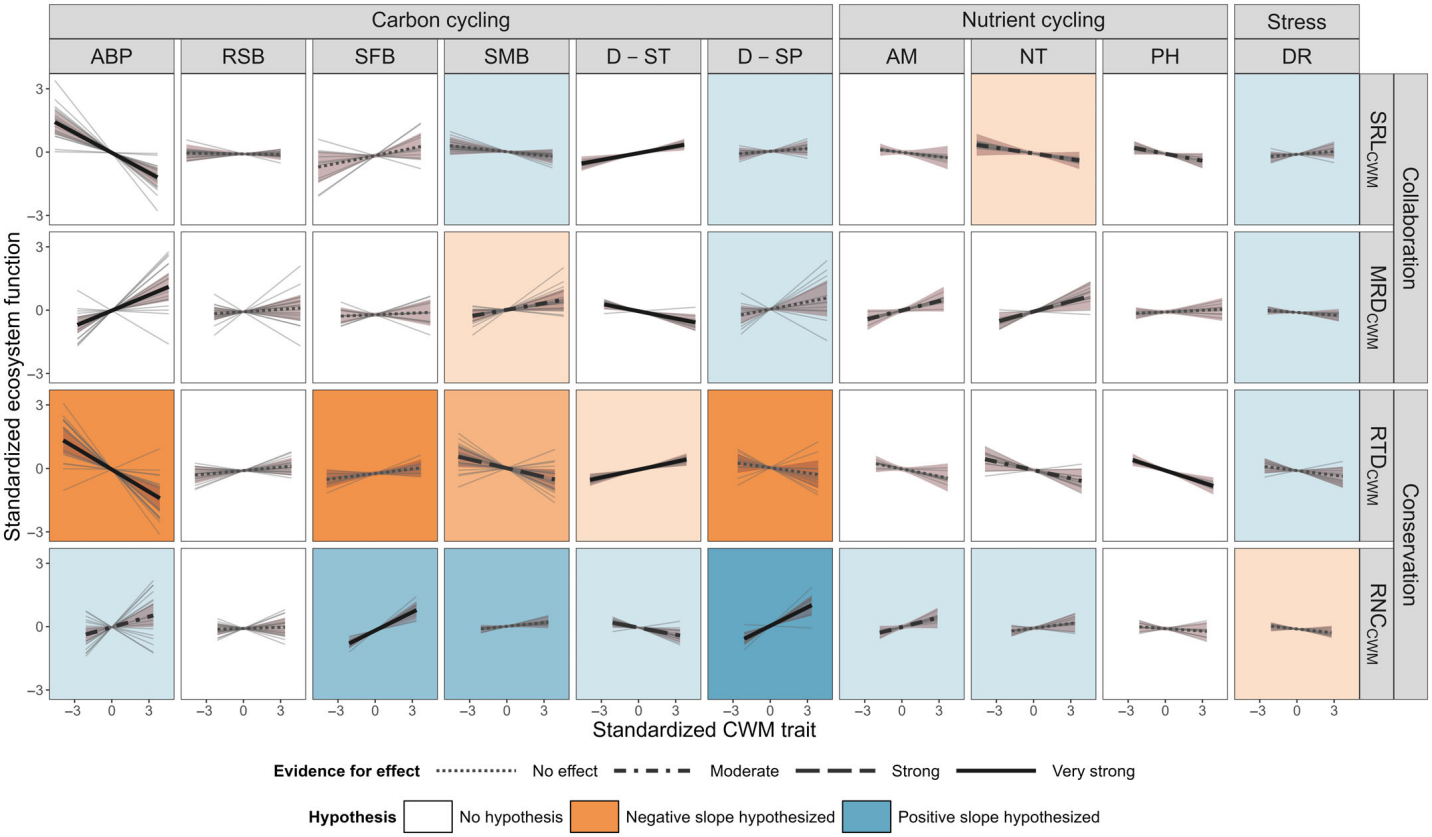
Results of Bayesian models examining the relationships between each ecosystem function and standardized community‐weighted mean root traits: specific root length (SRL_CWM_) and mean root diameter (MRD_CWM_) representing the collaboration gradient, and root tissue density (RTD_CWM_) and root nitrogen concentration (RNC_CWM_) representing the conservation gradient of the root economics space. Background colors indicate the hypothesized direction of effect based on our initial hypotheses (see Table 1), with color saturation reflecting the confidence in the expected direction. Black lines show the overall model slope, with line types representing the strength of evidence for an effect according to the probability of direction (PD), with PD ≤ 0.9 = no evidence of effect; 0.9 < PD < 0.95 = moderate evidence; 0.95 < PD < 0.975 = strong evidence; PD > 0.975 = very strong evidence. The shaded area depicts the 0.89 credible interval. Solid gray lines indicate the site‐specific slopes. Abbreviations of ecosystem functions: ABP, aboveground biomass production; AM, ammonification rate; DR, plant community drought resistance; D‐ST, decomposition of standard material; D‐SP, decomposition of plot‐specific litter; NT, nitrification rate; PH, soil phosphatase activity; RSB, root standing biomass; SFB, soil fauna biomass; SMB, soil microbial biomass.

Because the community‐level PCA did not fully reflect the species‐level root economics space, with RNC_CWM_ more closely related to MRD_CWM_ than RTD_CWM_, we chose to focus on individual root trait – ecosystem function relationships rather than using the PCs as an independent variable. We found that four of the 10 functions were related to SRL_CWM_ with at least moderate evidence for the effect (PD > 0.9), five with MRD_CWM_, five with RTD_CWM_, and five with RNC_CWM_ (Table 2, Fig. 2), respectively. For traits representing the conservation gradient (RTD_CWM_ – RNC_CWM_), we had *a priori* hypotheses for 14 out of the 20 trait–function relationships. For the collaboration gradient (SRL_CWM_ – MRD_CWM_), the literature allowed us to develop *a priori* hypotheses for only seven out of the 20 trait–function relationships. Contrary to our expectations, however, conservation and collaboration traits both appeared to be similarly important for trait–function relationships. We found similar numbers of relevant relationships across traits related to both the collaboration and the conservation axes, with nine relationships with traits of the collaboration gradient and ten with traits of the conservation gradient with at least moderate evidence for a direction of effect. However, our hypothesized direction was more often correct for traits of the conservation gradient (six correct hypotheses out of ten relevant relationships) than for traits of the collaboration gradient (one out of nine). For traits of the conservation gradient, we found two (out of ten) relationships were in the opposite direction than what we hypothesized (RTD_CWM_‐DST, RNC_CWM_‐DST, Fig. 2), while for traits of the collaboration gradient, one out of the nine relationships with evidence for a directional effect were hypothesized in the wrong direction (MRD_CWM_‐SMB). Overall, root traits explained relatively small amounts of variation in the single models (*R* ^2^ [0.008–0.200], Table 2).

Specifically, communities with higher SRL_CWM_ had lower aboveground biomass production (standardized estimate, estimate hereafter = −0.316), standard material was decomposed more quickly (estimate = 0.107), and soils tended to have lower nitrification rates (estimate = −0.089) and lower phosphatase activity (estimate = −0.110). Communities with high MRD_CWM_ had higher aboveground biomass production (estimate = 0.245), tended to have higher soil microbial biomass (estimate = 0.104), standard material was decomposed more slowly (estimate = −0.116), and soils tended to show higher rates of ammonification (estimate = 0.148) and had higher nitrification rates (estimate = 0.161). Communities with higher RTD_CWM_ had lower aboveground biomass production (estimate = −0.353) and lower soil microbial biomass (estimate = −0.139). They further had higher decomposition rates of standard material (estimate = 0.123) and soils tended to have lower nitrification rates (estimate = −0.132) as well as lower phosphatase activity (estimate = −0.189). Finally, communities with higher RNC_CWM_ tended to produce more aboveground biomass (estimate = 0.166), had higher soil fauna biomass (estimate = 0.291), standard material was decomposed more slowly (estimate = −0.112) but plot‐specific material was decomposed more quickly (estimate = 0.292), and soils tended to have higher ammonification rates (estimate = 0.1534). Root biomass (see Box 2) and drought resistance of the plant community were not related to any community‐weighted root trait.

Box 2 **Conceptual thinking on root biomass**

In our analysis, we include root standing biomass as a best available proxy for root productivity, a key function contributing to overall ecosystem productivity. However, it is of critical importance to recognize that root biomass itself may also be an important driver of other ecosystem functions (Lange *et al*., 2015) and as a scaler of the effects that individual root traits (MRD, SRL, RNC, RTD) have on other ecosystem functions. For example, when thinking about the decomposition of standard material (therefore unconfounded by the traits of the material), our hypotheses are based on the microbial community that assembles in the surroundings of the roots due to the root traits. While we expect that root biomass can alter the trait–functioning link, as higher plant biomass itself is associated with higher microbial biomass, root biomass could even act as a scaler for root traits, for example when root biomass disproportionately increases effects of litter quality. We currently do not specifically include these interactive effects of root biomass and traits but want to highlight that more work is needed to disentangle the context‐dependency of trait–functioning relationships in regard to root biomass.

## Discussion

We used a meta‐dataset to unearth community‐level root trait – ecosystem function relationships. We found that community‐weighted mean fine‐root trait values, in particular RNC (RNC_CWM_), did not conform with our previous findings associated with the RES established at the species level. We found evidence for the collaboration gradient (SRL_CWM_ and MRD_CWM_) at the community level but not the conservation gradient (RTD_CWM_ and RNC_CWM_). In spite of the lack of a RES at the community level, we found that the community‐weighted mean traits related to the conservation gradient were linked with 10 ecosystem functions, especially those related to carbon cycling. Similarly, traits of the collaboration gradient were related to nine ecosystem functions. Of the 10 ecosystem functions we examined, only root biomass (Box 2) and drought resistance were not correlated with any of the root traits.

### The RES at the community level

When including the 317 species with complete trait data, the PCA of the root traits resembled the RES of Bergmann *et al*. (2020) with orthogonal coordination of the collaboration gradient, formed by SRL and MRD, and the conservation gradient, formed by RTD and RNC (Fig. 1a). However, at the community level, we found strong evidence for a collaboration gradient but not a conservation gradient. RNC_CWM_ loaded on both the first and third components rather than on the second, with a positive bivariate correlation between RNC_CWM_ and MRD_CWM_ that is not present at the species level (Table S5). Community‐level patterns like those observed here may occur in systems with a high relative abundance of legumes, which tend to have high RNC due to the presence of nitrogen‐fixing rhizobia, regardless of their other traits. A divergent role of RNC_CWM_ is common in the literature (Sweeney *et al*., 2021; Xia *et al*., 2021; Lachaise *et al*., 2022), and our RES partially aligns with the community‐level analysis of Lachaise *et al*. (2022), who also found that RNC_CWM_ shifted almost entirely to PC1 at the community level.

In the 810 communities we examined, there could be several reasons why the community‐level RES differed from our expectations, which were based on our previous observations at the species level. First, species with specific traits (e.g. association with nitrogen‐fixing rhizobia as described above) may be more or less abundant in a community because of the abiotic and biotic conditions of a given ecosystem and climate, as well as the soil conditions of a plot location (Anderegg, 2023). That is, one of the characteristics of communities is that they are not subject to the same limitations as species traits and may not have to adhere to the same trade‐offs. Second, we calculated our community‐weighted means using aboveground community composition and abundance, which may not accurately reflect either belowground community composition or the associated abundance of fine roots of a given species. This mismatch may decrease our capacity to accurately reflect the community trait space belowground and may especially affect our results when belowground and aboveground dynamics are not matched (Hiiesalu *et al*., 2012; Barry *et al*., 2019; Martin‐Guay *et al*., 2020; Ottaviani *et al*., 2020). Third, we use data on species‐level traits and then calculate community‐weighted means. This approach ignores intraspecific trait variation reflecting adaptations to local conditions, including the presence of other species. Measuring this plasticity requires *in situ* trait measurements and may reveal community‐level trait coordination that is more similar to the species‐based RES. Fourth, biases in the availability of trait data may decrease the likelihood that a community‐level RES is present. We eliminated plots from our analysis where complete trait data were available for a subset of species representing < 80% of the community relative abundance (*c*. 58.7% of plots). Rare species are less likely to have complete trait data available, and therefore sites with high species richness were often excluded in our analysis. By contrast, sites whose communities contain a small actual or effective number of species may not demonstrate a community‐level RES because when we have fewer species in a community or when many communities are dominated by the same species, these are more likely to represent extremes of the trait space or alter trait coordination toward their dominant traits. In our dataset, sites like the Kreinitz Biodiversity Experiment, where the community‐level PCA did not represent the RES (Fig. S4), had only six species maximum.

### Root trait – Ecosystem function relationships

Based on our initial literature search (Table 1), we expected traits that fall on the conservation gradient (i.e. RTD_CWM_ and RNC_CWM_) to be more closely related to ecosystem functions than those that fall on the collaboration gradient (i.e. SRL_CWM_ and MRD_CWM_). This expectation relied on our capacity to develop hypotheses for trait‐ecosystem function relationships from the literature. These differences in our capacity and confidence in expected relationships between traits and functions on the conservation gradient vs the collaboration gradient may reflect a bias in the literature surrounding trait–function relationships. The conservation gradient is well studied aboveground (Wright *et al*., 2004; Reich, 2014; Díaz *et al*., 2016) and has been expanded to include belowground plant traits since at least 2013 (Chen *et al*., 2013; Kong *et al*., 2014). This density of information made it easier to develop hypotheses for the conservation gradient belowground but also to extrapolate from aboveground dynamics across this gradient. The collaboration gradient, however, has only been formalized in the literature more recently (2020). This relative novelty may limit our capacity to anticipate how these belowground traits alter function, at least based on historical literature.

All but two of the functions investigated (root standing biomass and drought resistance) were related to at least one fine‐root trait of the RES (moderate evidence for an effect in 19 out of 40 individual relationships; Table 2; Figs 2, S5). While this plethora of relationships indicates that trait‐ecosystem function relationships may be common, most of these individual relationships explained a relatively low proportion of variance (Table 2). The magnitude of explanatory power is comparable to similar analyses when they are found (van der Plas *et al*., 2020). Functions that were more strongly correlated may rely more on resource acquisition by fine roots, which is reflected by the RES. For example, aboveground biomass production is directly related to resource use and uptake, which is determined largely by the fine roots characterized by the RES. Drought resistance, however, may be more related to hydraulic traits or the capacity of the roots to reach deeper water resources than to the resource acquisition traits incorporated in the RES (Laughlin *et al*., 2023). Some functions (e.g. root biomass and drought resistance) are also more derived proxies for actual ecosystem functions than many of our other proxies used in this study, which may explain their weak link with root functional traits (see Box 2 for discussion of root biomass in particular). This variation may also reflect our use of aboveground community composition to calculate our community‐weighted mean root traits. Many of these composition measures are strongly correlated with, for example, aboveground biomass.

Functions related to carbon cycling, including aboveground biomass production, soil fauna biomass, soil microbial biomass, as well as decomposition of standard and plot‐specific litter, were largely correlated with traits of the conservation gradient. This is in line with previous studies (Wardle *et al*., 2004; Da *et al*., 2023; Jimoh *et al*., 2024) and matches our mechanistic understanding of the conservation gradient as a trade‐off in resource use (Reich, 2014). The role of traits of the collaboration gradient in carbon dynamics is much less investigated, and as a result, we had less evidence with which to build our hypotheses. However, we found correlations of collaboration gradient traits with functions related to carbon cycling, comparable in strength and frequency to those of conservation gradient traits (Table 2; Fig. 2). This link may be due to the differences in root anatomy and mycorrhizal colonization that give the collaboration gradient its name. For example, we unexpectedly found moderate evidence for a positive relationship between MRD_CWM_ and soil microbial biomass (Fig. 2). Recent evidence suggests that thicker roots are associated with higher rhizodeposition (Folacher *et al*., 2024) and exudation (Williams *et al*., 2022), which is then paired with higher mycorrhizal colonization, potentially resulting in higher soil microbial biomass.

*A priori*, we had fewer hypotheses for ecosystem functions related to nutrient cycling than for carbon cycling since specific soil microbial processes have been rarely studied in the context of interspecific variation in root traits and because factors other than plants exert strong controls over soil nutrient cycling, including edaphic conditions such as soil texture, moisture, and oxygen content, as well as microbial community composition and activity, though we controlled for some of this variation in our statistical framework. We found that ammonification and nitrification rates were associated with traits of both the collaboration and conservation gradient. For example, on the collaboration gradient, nitrification increased in communities with higher MRD_CWM_ and decreased, though by a small amount, in communities with higher SRL_CWM_. Both of these patterns may be linked to the higher soil microbial biomass in higher MRD_CWM_ communities (Fig. 2). Since this applied to nitrification – but not to ammonification – greater oxygen availability in soil resulting from larger pores induced by thick roots might also explain increased nitrification (Bollmann & Conrad, 1998; Bodner *et al*., 2014). On the conservation gradient, communities with high RTD_CWM_ had low nitrification rates, which may be less due to the roots than to the general relationship between RTD_CWM_ and nutrient availability. High RTD_CWM_ communities tend to occur on low nitrogen sites, leading to less overall N available for nitrification and low nitrifier abundance (Table 1; Legay *et al*., 2014).

In general, our community‐level results differ from previous studies which looked at broad ranges of trait–function relationships. For example, van der Plas *et al*. (2020) examined trait–functioning relationships for two of our four traits and seven of our 10 ecosystem functions for one experimental grassland site. Of the possible 14 overlapping significant relationships, only one was the same in our analysis. This disparity may be due to our inclusion of observational systems in our dataset in addition to experimental manipulations. Biodiversity experiments often try to minimize environmental variation (Hooper *et al*., 2005; Tilman *et al*., 2014; Jochum *et al*., 2020). Yet environmental variation drives changes in functional traits themselves, the relative abundance of species with certain functional traits, and ecosystem functions simultaneously. This major difference between our results and others highlights some potential limitations for using biodiversity experiments to explore trait–function relationships. Unmanipulated community assembly may increase the likelihood that traits and functions are related to each other, although at the same time it makes conclusions about causality more challenging.

### Conclusion

This study highlights three important lessons in matching community‐weighted plant functional traits to ecosystem functions. First, our capacity to understand the universality of trait–function relationships may be limited by general biases in the publicly available root trait databases. Observational systems, where trait–function relationships may be most likely, are often excluded from analyses because of their higher diversity, including rare species, for which we may not have adequate trait measurements. This bias is likely to be especially prominent outside of North America and western Europe, where there are even fewer trait measurements in common databases (Kattge *et al*., 2020). Second, our analysis highlights a need to think critically about when we may expect communities to conform to ideal trait distributions. Communities with small species pools and high dominance of individual species did not appear to conform to patterns we previously observed in species‐level trait spectra. These relatively common patterns (low species number and high dominance) may limit the capacity of species‐level trait spectra to explain ecosystem functions. However, at the community level, the collaboration gradient of the root economics space may be more consistent than the conservation gradient across systems. Further, one strength of communities is that they are not subject to the same trade‐offs as species and therefore may not be expected to adhere to species‐level patterns. Finally, our analysis is unable to examine the consequences of changes in trait expression in communities because we use species‐level traits to calculate community‐weighted means rather than traits measured in the communities themselves.

In our study, each individual functional trait could only explain a small amount of variance in ecosystem functions. However, as suggested by Freschet *et al*. (2021b) and Streit & Bellwood (2023), matching multiple traits to functions with clear direct conceptual links increased our capacity to understand links. We showed that fine‐root traits of both the conservation (RTD_CWM_ and RNC_CWM_) and the collaboration (SRL_CWM_ and MRD_CWM_) axes correlated with key ecosystem functions across a range of experimental and observational sites in grasslands and forests. Further, the majority of the functions that we examined were related to multiple independent traits. Our findings indicate that shifts in the relative abundance of commonly measured traits can alter important ecosystem functions such as carbon cycling. The interrelatedness of these trait–function relationships suggests that changes in the communities' traits are likely to have complex and interacting effects on ecosystem functions. This interrelatedness highlights a need for experiments that directly manipulate the traits of the community, for example, by designing experimental plant communities with species combinations that form two orthogonal gradients in collaboration and conservation traits.

## Competing interests

Colleen M. Iversen is an author on this paper and an Editor at *New Phytologist*. Thomas W. Kuyper was previously a member of the Board of Advisors of *New Phytologist*.

## Author contributions

Conceptualization and workshop participation: KEB, JH, AW, JB, HB, GF, CMI, TWK, DCL, MLM, CR, FvdP, JvR, LM. Data contribution: AW, DCL, JvR, HA, NE, JA, CN, LM, YO, WW, RW. Analysis: KEB, JH. Writing – original draft: KEB, JH, AW, LM. Writing – review and editing: all authors. Funding acquisition: AW, LM. KEB and JH contributed equally to this work.

## Disclaimer

The New Phytologist Foundation remains neutral with regard to jurisdictional claims in maps and in any institutional affiliations.

## Supporting information


**Fig. S1** PCA of community‐weighted traits, standardized within each project site and ecosystem, without a cutoff for trait data availability.
**Fig. S2** Posterior predictive checks of Bayesian hierarchical models.
**Fig. S3** Ridge plot of posterior distributions of trait effects on ecosystem functions across trait–function combinations.
**Fig. S4** Separate PCA at the community level per project and ecosystem.
**Fig. S5** Trait–functioning relationships based on linear Bayesian hierarchical models including raw data points.
**Table S1** Data sources.
**Table S2** Proxies of ecosystem functions from the individual datasets.
**Table S3** Number of plots per function for each project and ecosystem without a cutoff for trait data availability.
**Table S4** Number of plots per function for each project and ecosystem with a minimum of trait data available for 80% of the plant community.
**Table S5** Pairwise Pearson correlations between the four root traits at the species and community level.
Please note: Wiley is not responsible for the content or functionality of any Supporting Information supplied by the authors. Any queries (other than missing material) should be directed to the *New Phytologist* Central Office.

## Data Availability

The data and code needed to reproduce the analyses of the study are available via Zenodo at https://doi.org/10.5281/zenodo.15355986. The individual datasets included in the study are listed in Table S1.
