# Supplementary material for: Rooting for function: community‐level fine‐root traits relate to many ecosystem functions
Source: New Phytol. 2025 Oct 3;248(6):3221–39. doi: 10.1111/nph.70606 (PMC12630466; doi:10.1111/nph.70606)
Supplement: Supplementary file 1 — Fig. S1 PCA of community‐weighted traits, standardized within each project site and ecosystem, without a cutoff for trait data availability. Fig. S2 Posterior predictive checks of Bayesian hierarchical models. Fig. S3 Ridge plot of posterior distributions of trait effects on ecosystem functions across trait–function combinations. Fig. S4 Separate PCA at the community level per project and ecosystem. Fig. S5 Trait–functioning relationships based on linear Bayesian hierarchical models including raw data points. Table S1 Data sources. Table S2 Proxies of ecosystem functions from the individual datasets. Table S3 Number of plots per function for each project and ecosystem without a cutoff for trait data availability. Table S4 Number of plots per function for each project and ecosystem with a minimum of trait data available for 80% of the plant community. Table S5 Pairwise Pearson correlations between the four root traits at the species and community level. Please note: Wiley is not responsible for the content or functionality of any Supporting Information supplied by the authors. Any queries (other than missing material) should be directed to the New Phytologist Central Office. [file NPH-248-3221-s001.pdf]

New Phytologist

***New Phytologist* Supporting Information**

Article title: Rooting for function: Community-level fine-root traits relate to many ecosystem functions

Authors: Kathryn E. Barry, Justus Hennecke, Alexandra Weigelt, Joana Bergmann, Helge Bruelheide, Grégoire T. Freschet, Colleen M. Iversen, Thomas W. Kuyper, Daniel C. Laughlin, M. Luke McCormack, Catherine Roumet, Fons van der Plas, Jasper van Ruijven, Rachel Wijsmuller, Harald Auge, Nico Eisenhauer, Josephine Haase, Charles Nock, Yvonne Oelmann, Wolfgang Wilcke and Liesje Mommer

Article acceptance date: 2 September 2025

The following Supporting Information is available for this article:

Fig. S1: PCA of community-weighted traits, standardized within each project site and ecosystem, without a cutoff for trait data availability.

Fig. S2: Posterior predictive checks of Bayesian hierarchical models.

Fig. S3: Ridge plot of posterior distributions of trait effects on ecosystem functions across trait–function combinations.

Fig. S4: Separate PCA at the community-level per project and ecosystem.

Fig. S5: Trait-functioning relationships based on linear Bayesian hierarchical models including raw data points.

Table S1: Data sources.

Table S2: Proxies of ecosystem functions from the individual datasets.

Table S3: Number of plots per function for each project and ecosystem without a cutoff for trait data availability.

Table S4: Number of plots per function for each project and ecosystem with a minimum trait data available for 80% of the plant community.

Table S5: Pairwise Pearson correlations between the four root traits at the species- and community level.

Fig. S1: PCA of community-weighted traits, standardized within each project site and ecosystem, without a cutoff for trait data availability (n = 1963).

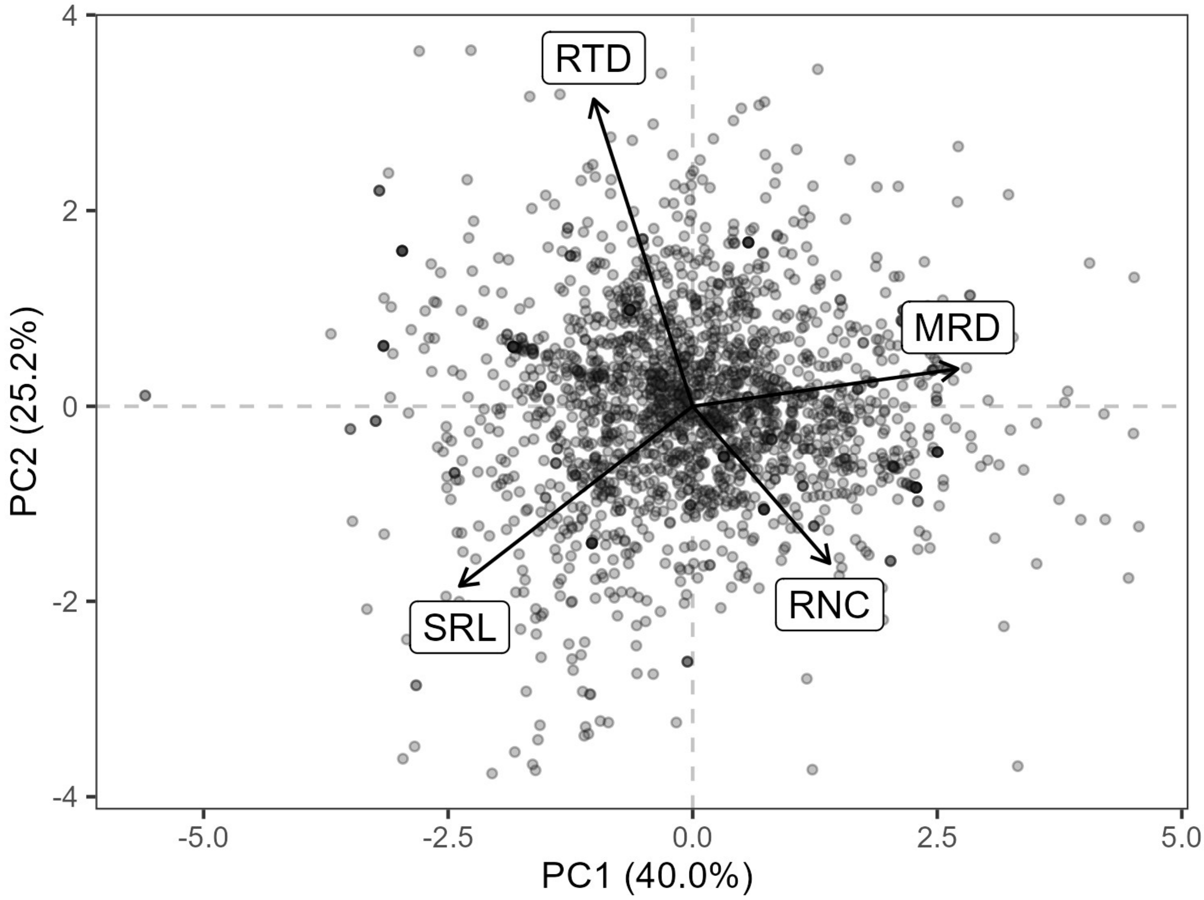

Fig. S2: Posterior predictive checks of Bayesian hierarchical models. SRL, specific root length; MRD, mean root diameter; RTD, root tissue density; RNC, root nitrogen content; ABP, aboveground biomass production; RSB, root standing biomass; SFB, soil fauna biomass; SMB, soil microbial biomass; D-ST, decomposition of standard material; D-SP, decomposition of plot-specific litter; AM, ammonification rate; NT, nitrification rate; PH, soil phosphatase activity; DR, plant community drought resistance.

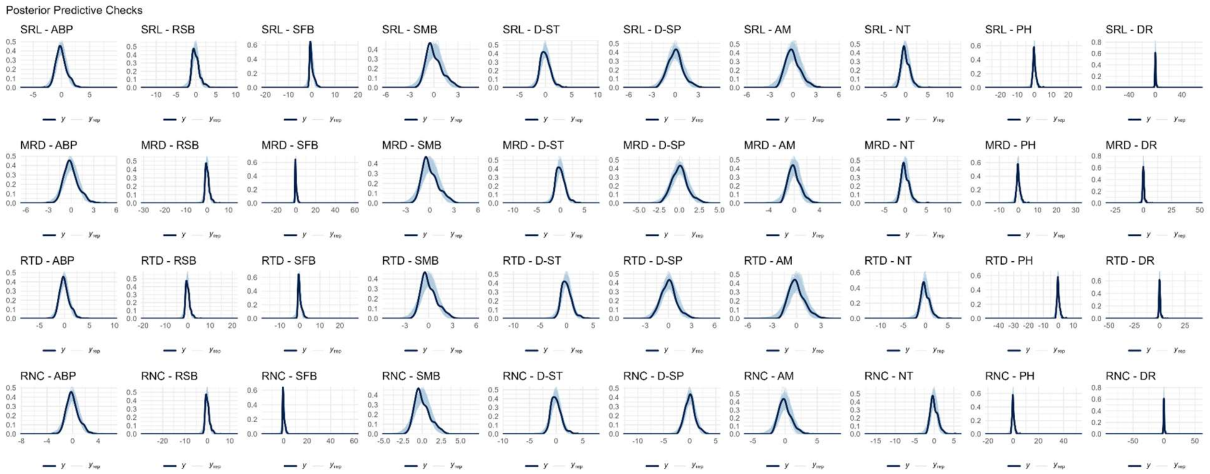

Fig. S3: Ridge plot of posterior distributions of trait effects on ecosystem functions across trait–function combinations. Each distribution represents the posterior uncertainty around the estimated slope for a specific trait–function pair, based on hierarchical Bayesian models. Distributions centered away from zero indicate stronger evidence for a directional effect. SRL, specific root length; MRD, mean root diameter; RTD, root tissue density; RNC, root nitrogen content; ABP, aboveground biomass production; RSB, root standing biomass; SFB, soil fauna biomass; SMB, soil microbial biomass; D-ST, decomposition of standard material; D-SP, decomposition of plot-specific litter; AM, ammonification rate; NT, nitrification rate; PH, soil phosphatase activity; DR, plant community drought resistance.

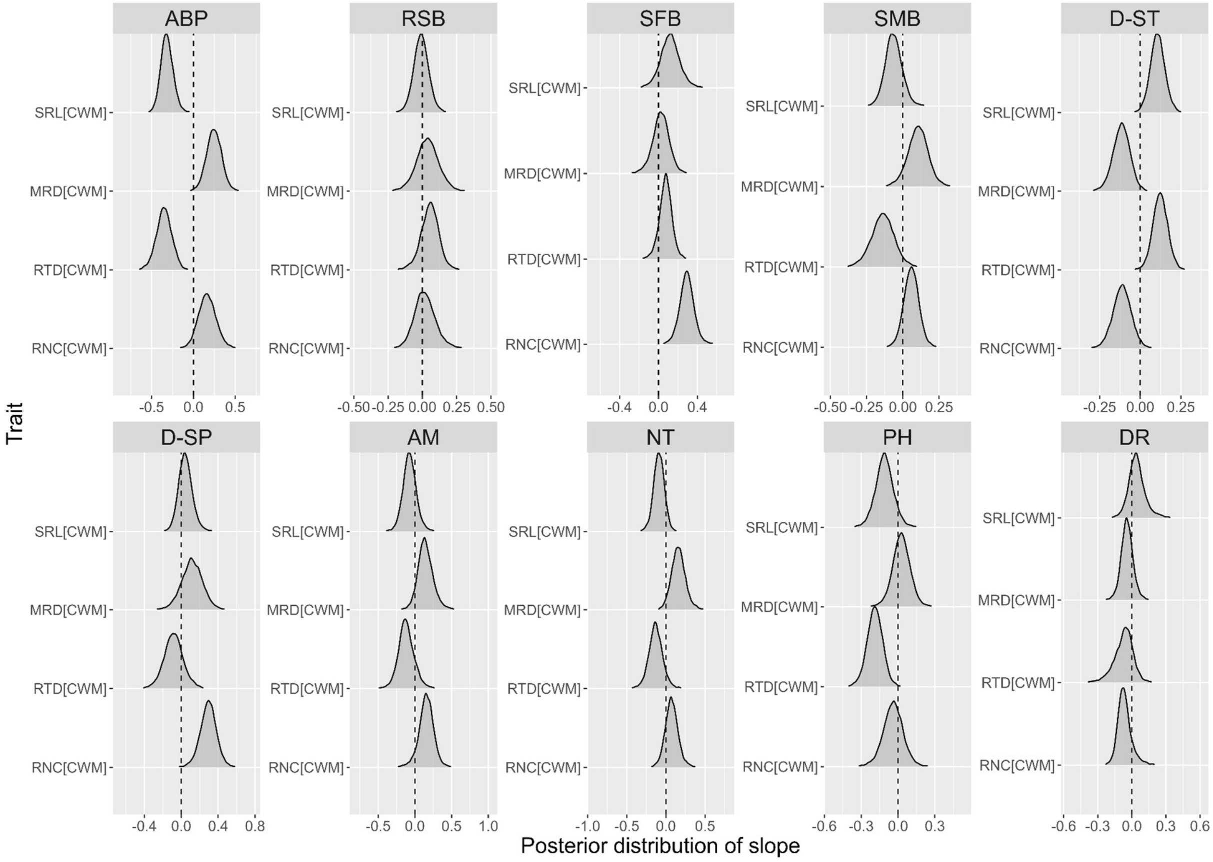

New Phytologist

Fig. S4: Separate PCA plots at the community-level per project and ecosystem. Only plant communities/plots of which at least 80% of the plant abundance have complete trait data are included.

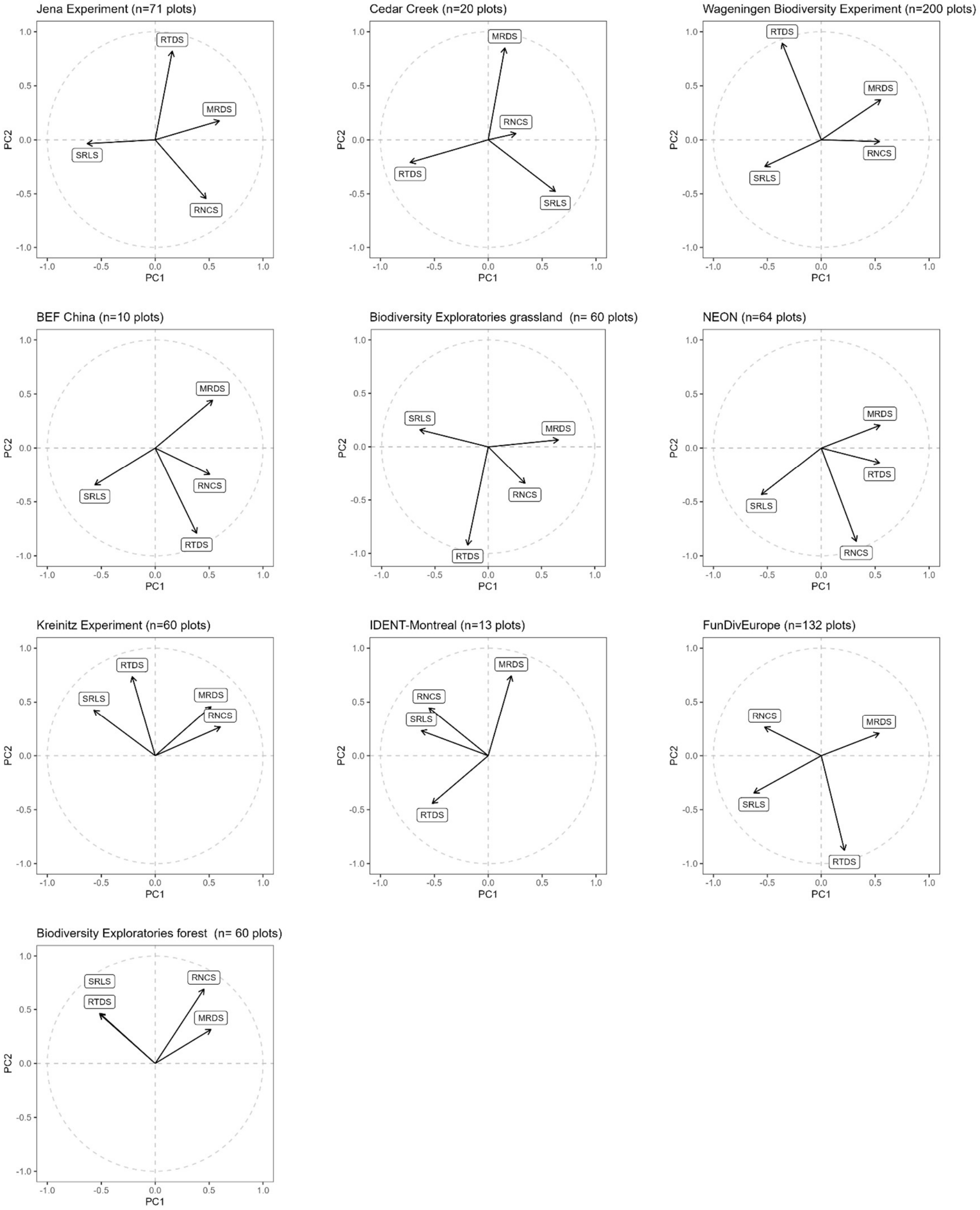

Fig. S5: Results of Bayesian models examining the relationships between each ecosystem function and standardized community-weighted mean root traits: specific root length (SRL_{CWM}) and mean root diameter (MRD_{CWM}) representing the collaboration gradient, and root tissue density (RTD_{CWM}) and root nitrogen concentration (RNC_{CWM}) representing the conservation gradient of the root economics space. Blue points are raw data point and black lines show the overall model slope, with line types representing the strength of evidence for an effect according to the probability of direction (PD), with PD≤0.9 = no evidence of effect; 0.9<PD<0.95 = moderate evidence; 0.95<PD<0.975 = strong evidence; PD >0.975 = very strong evidence. The shaded area depicts the 0.89 credible interval. Solid grey lines indicate the site-specific slopes. Abbreviations of ecosystem functions: ABP, aboveground biomass production; RSB, root standing biomass; SFB, soil fauna biomass; SMB, soil microbial biomass; D-ST, decomposition of standard material; D-SP, decomposition of plot-specific litter; AM, ammonification rate; NT, nitrification rate; PH, soil phosphatase activity; DR, plant community drought resistance.

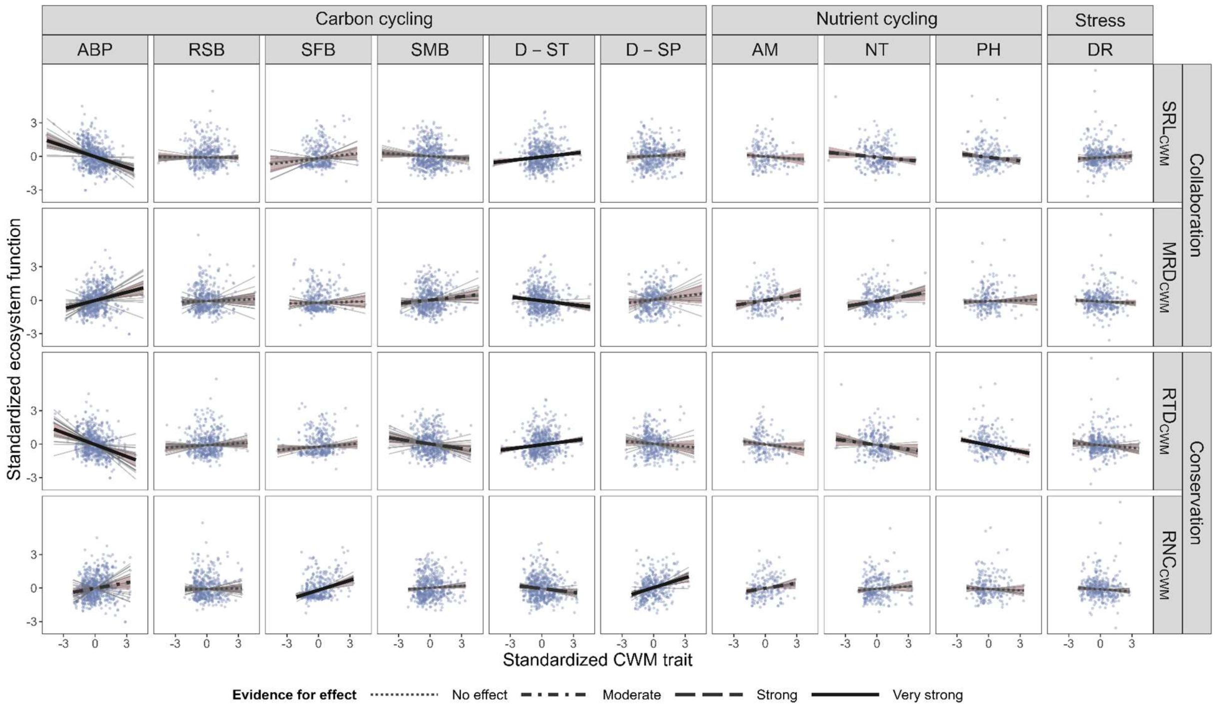

Table S1: Data sources for the community composition and ecosystem function data in the synthesized dataset.

Project, Location	Location	Biome	Data source
BEF China	China	subtropical	Schuldt *et al.* (2018)
Biodiversity Exploratories	Germany	temperate	data from Solly *et al.* (2014), Felipe-Lucia *et al.* (2018)<br>and datasets:<br>31175 (Hinderling & Ralph Bolliger, 2023), 18268 (Schall et al., 2017), 27087 (Schenk et al., 2023), 20251 (Kandeler et al., 2021), 24367 (Allan et al., 2018), 21686 (Bluhm & Scheu, 2018), 20926 (Rueß, 2021), 14448 (E. Solly et al., 2023) from https://www.bexis.uni-jena.de/
Cedar Creek	US	temperate	data from Isbell *et al.* (2015), and datasets: Tilman (2018, 2024a, 2024b), Reich (2021, 2024b, 2024a)
FunDivEurope	Europe	temperate	data from Grossiord *et al.* (2014), van der Plas *et al.* (2016), Ratcliffe *et al.* (2017), and additional data
IDENT-Montreal	Canada	temperate	Martin-Guay *et al.* (2020)
Jena Experiment	Germany	temperate	data from Hacker *et al.* (2015), Chen *et al.* (2017), Lama *et al.* (2020), van der Plas *et al.* (2020) and dataset 78 (Eisenhauer, 2020) from https://jexis.idiv.de/
Kreinitz Experiment	Germany	temperate	Gottschall *et al.* (2019) and additional data
NEON	US	boreal, temperate, subtropical, tropical	National Ecological Observatory Network (NEON) (2024a, 2024d, 2024b, 2024c)
Wageningen Biodiversity Experiments	the Netherlands	temperate	Cong *et al.* (2014) and unpublished data

Table S2: Proxies of the ecosystem functions in the individual datasets and their original units.

	BEF China	Biodiversity Exploratories Forests	Biodiversity Exploratories Grasslands	Cedar Creek	FunDivEurope	IDENT-Montreal	Jena Experiment	Kreinitz Experiment	NEON Forests
**Metric of community composition**	Number of plant individuals >1m	Basal area at breast height [cm^2]	Cover (%)	Aboveground dry biomass [g m^{-2}]	Basal area at breast height [m^2]	Aboveground dry biomass based on tree allometry [g plot^{-1}]	Aboveground dry biomass [g m^{-2}]	Basal area at 10cm height [cm^2]	Cover (%)
**Aboveground biomass production**	Basal area increment of woody plants at breast height [m^2 ha^{-1} a^{-1}]	Mean annual increment in wood volume [m^3 ha^{-1} a^{-1}]	Aboveground dry biomass [g m^{-2}]	Aboveground dry biomass [g m^{-2}]	Aboveground productivity of trees based on annual radius growth and tree allometry [Mg C ha^{-1} a^{-1}]	-	Aboveground dry mass of target species [g m^{-2}]	Mean annual basal area increment at 10cm height [cm^2 a^{-1}]	-
**Root standing biomass**	-	Dry root biomass of fine and coarse roots within the upper 10 cm of the mineral soil [g (2747.5 cm^3)$^{-1}$]	Dry root biomass of fine and coarse roots within the upper 10 cm of the mineral soil [g (2747.5 cm^3)$^{-1}$	Root dry biomass upper 30cm of soil [g m^{-2}]	Fine root dry biomass upper 10cm or upper 20cm of soil [g m^{-2} a^{-1}]	Dry root biomass of fine and coarse roots [g plot^{-1}]	Dry root biomass of fine and coarse roots upper 30cm of soil [g m^{-2}]	-	Root dry biomass of roots smaller than 10mm in diameter in the upper 30 cm of soil [g m^{-2}]
**Soil fauna biomass**	-	Fresh earthworm biomass [g (0.25 m^2)$^{-1}$]	-	-	Fresh earthworm biomass [g m^{-2}]	-	Fresh earthworm biomass [g m^{-2}]	Fresh earthworm biomass [g m^{-2}]	-
**Soil microbial biomass**	Sum of PLFA [nmol g dry soil^{-1}]	Sum of PLFA [nmol g dry soil^{-1}]	Sum of microbial PLFA FAMEs [nmol FAME g dry soil^{-1}]	Soil microbial carbon biomass [µg Cmic g dry soil^{-1}]	Soil microbial biomass carbon [mg kg dry soil^{-1}]	-	Soil microbial carbon biomass [µg Cmic g dry soil^{-1}]	soil basal respiration [µl O2 hr^{-1} g dry soil^{-1}]	sum of PLFA concentration [µg g dry soil^{-1}]

**Decomposition - standard material**	Leaf decomposition constant for Schima superba [exponential decay coefficient k]	Mass loss after 6 months of decomposition [%]	Mass loss after 6 months of decomposition [%]	-	Mass loss of standard wood litter [%]	-	Mass loss of fine roots [%]	Wood mass loss [%]	-
**Decomposition - plot-specific material**	Leaf decomposition constant [exponential decay coefficient k]	-	Mean daily decomposition rate [%]	-	Mass loss of plot-specific leaf litter [%]	-	Mass loss of plot-specific fine roots [%]	-	-
**Ammonification**	Gross ammonification rate [mg kg^{-1} d^{-1}]	-	-	Net nitrogen mineralization rate [mg kg^{-1} d^{-1}]	-	-	Gross N mineralization [µg N g^{-1} d^{-1}]	Gross N mineralization [µmol g^{-1} d^{-1}]	Net nitrogen mineralization rate [µg N·g^{-1}·d^{-1})
**Nitrification**	Gross nitrification rate [mg kg^{-1} d^{-1}]	Potential nitrification rate, as nitrite accumulation over time [ng NO_2 g dry $soil^{-1}$ h^{-1}]	Potential nitrification rate, as nitrite accumulation over time (ng [ng NO_2 g dry $soil^{-1}$ h^{-1}]	-	-	-		Gross nitrification [µmol g^{-1} d^{-1}]	Net nitrogen nitrification rate [µg N·g^{-1}·d^{-1}]
**Phosphatase activity**	Acid Phosphatase activity [nmol g dry $soil^{-1}$ h^{-1}]	-	Activity measured using fluorescent 4-methylumbelliferone substrates (4-MUF) [nmol MUF g^{-1} h^{-1}]	Acid phosphatase activity [nmol g dry $soil^{-1}$ h^{-1})	Acid phosphatase activity [nmol g dry $soil^{-1}$ h^{-1}]	-	Phosphodiesterase activity [µg p-Nitrophenol g dry $soil^{-1}$ h^{-1}]	-	-

**Drought resistance of plant community**	-	-	-	Drought resistance index calculated as ecosystem productivity nondrought year/(ecosystem productivity in drought year - ecosystem productivity nondrought year)	Difference in δ13C in wood between wet and dry year	-	Drought resistance calculated as (aboveground biomass drought year – aboveground biomass pre-drought year) / aboveground biomass pre-drought year	-	-

Table S3: Number of plots (plant communities) per function for each project and ecosystem without a cutoff for trait data availability.

Project	Ecosystem type	Project type	Aboveground biomass production	Standing biomass	Soil fauna biomass	Soil microbial biomass	Decomposition - standard material	Decomposition - plot-specific	Ammonification	Nitrification	Phosphatase activity	Drought resistance of plant community
BEF China	forest	observational	27	0	0	27	27	27	27	27	27	0
Biodiversity Exploratories	forest	observational	150	150	134	150	135	0	0	150	0	0
Biodiversity Exploratories	grassland	observational	149	149	0	149	136	148	0	148	149	0
Cedar Creek	grassland	experimental	207	213	0	21	0	0	58	0	21	207
FunDivEurope	forest	observational	209	208	209	206	209	204	0	0	64	185
IDENT-Montreal	forest	experimental	0	96	0	0	0	0	0	0	0	0
Jena Experiment	grassland	experimental	78	70	45	70	80	80	78	0	80	75
Kreinitz Experiment	forest	experimental	95	0	96	96	95	0	12	12	0	0
NEON	forest	observational	0	68	0	191	0	0	206	206	0	0
NEON	grassland	observational	0	21	0	73	0	0	73	73	0	0
Wageningen Biodiversity Experiment	grassland	experimental	201	0	0	0	97	96	68	0	0	201
Total	Total	Total	1116	975	484	983	779	555	522	616	341	668

Table S4: Number of plots (plant communities) per function for each project and ecosystem with a minimum trait data available for 80% of the plant community.

Project	Ecosystem type	Project type	Aboveground biomass production	Standing biomass	Soil fauna biomass	Soil microbial biomass	Decomposition - standard material	Decomposition - plot-specific	Ammonification	Nitrification	Phosphatase activity	Drought resistance of plant community
BEF China	forest	observational	10	0	0	10	10	10	10	10	10	0
Biodiversity Exploratories	forest	observational	137	137	124	137	123	0	0	137	0	0
Biodiversity Exploratories	grassland	observational	103	103	0	103	97	103	0	103	103	0
Cedar Creek	grassland	experimental	11	11	0	9	0	0	11	0	9	10
FunDivEurope	forest	observational	132	131	132	129	132	129	0	0	47	123
IDENT-Montreal	forest	experimental	0	13	0	0	0	0	0	0	0	0
Jena Experiment	grassland	experimental	66	61	37	60	69	69	67	0	69	65
Kreinitz Experiment	forest	experimental	59	0	60	60	60	0	8	8	0	0
NEON	forest	observational	0	2	0	1	0	0	5	5	0	0
NEON	grassland	observational	0	5	0	15	0	0	15	15	0	0
Wageningen Biodiversity Experiment	grassland	experimental	200	0	0	0	96	95	67	0	0	200
Total			718	463	353	524	587	406	183	278	238	398

Table S5: Pairwise pearson correlation coefficient of the four root traits at the species- and community level. Only complete cases and communities with a minimum trait data available for 80% of the plant community were considered (species-level: *n* = 317, community-level: *n* = 810).

Traits	Species-level correlation	Community-level correlation
MRD - SRL	-0.38***	-0.65***
MRD - RNC	0.02	0.45***
MRD - RTD	0.01	-0.12***
SRL - RNC	-0.02	-0.27***
SRL - RTD	-0.32***	0.24***
RTD - RNC	-0.22***	-0.23***

References:

Allan, E., Felipe-Lucia, M., Ammer, C., Bauhus, J., Fischer, M., Nauss, T., Polle, A., Schall, P., Schloter, M., Schöning, I., Schrumpf, M., Sorkau, E., Tschapka, M., Weisser, W., Wubet, T., & Blüthgen, N. (2018). *Raw data of forest attributes of forest EPs of the Exploratories project used in „Multiple forest attributes underpin the supply of multiple ecosystem services" (ID 24367)* [Dataset]. Biodiversity Exploratories Information System. https://www.bexis.uni-jena.de

Bluhm, S., & Scheu, S. (2018). *The earthworm biomass of all forest EPs from spring 2011* [Dataset]. Biodiversity Exploratories Information System. https://www.bexis.uni-jena.de/ddm/data/Showdata/21686?version=2

Chen, H., Mommer, L., van Ruijven, J., de Kroon, H., Fischer, C., Gessler, A., Hildebrandt, A., Scherer-Lorenzen, M., Wirth, C., & Weigelt, A. (2017). Plant species richness negatively affects root decomposition in grasslands. *Journal of Ecology*, *105*(1), 209–218. https://doi.org/10.1111/1365-2745.12650

Cong, W.-F., van Ruijven, J., Mommer, L., De Deyn, G. B., Berendse, F., & Hoffland, E. (2014). Plant species richness promotes soil carbon and nitrogen stocks in grasslands without legumes. *Journal of Ecology*, *102*(5), 1163–1170. https://doi.org/10.1111/1365-2745.12280

Eisenhauer, N. (2020). *Soil microbial respiration and biomass_main_2003 to 2020* [Dataset]. https://jexis.idiv.de/ddm/data/Showdata/78

Felipe-Lucia, M. R., Soliveres, S., Penone, C., Manning, P., van der Plas, F., Boch, S., Prati, D., Ammer, C., Schall, P., Gossner, M. M., Bauhus, J., Buscot, F., Blaser, S., Blüthgen, N., de Frutos, A., Ehbrecht, M., Frank, K., Goldmann, K., Hänsel, F., … Allan, E. (2018).

Multiple forest attributes underpin the supply of multiple ecosystem services. *Nature Communications*, *9*(1), 4839. https://doi.org/10.1038/s41467-018-07082-4

Gottschall, F., Davids, S., Newiger-Dous, T. E., Auge, H., Cesarz, S., & Eisenhauer, N. (2019). Tree species identity determines wood decomposition via microclimatic effects. *Ecology and Evolution*, *9*(21), 12113–12127. https://doi.org/10.1002/ece3.5665

Grossiord, C., Granier, A., Ratcliffe, S., Bouriaud, O., Bruelheide, H., Chećko, E., Forrester, D. I., Dawud, S. M., Finér, L., Pollastrini, M., Scherer-Lorenzen, M., Valladares, F., Bonal, D., & Gessler, A. (2014). Tree diversity does not always improve resistance of forest ecosystems to drought. *Proceedings of the National Academy of Sciences*, *111*(41), 14812–14815. https://doi.org/10.1073/pnas.1411970111

Hacker, N., Ebeling, A., Gessler, A., Gleixner, G., González Macé, O., de Kroon, H., Lange, M., Mommer, L., Eisenhauer, N., Ravenek, J., Scheu, S., Weigelt, A., Wagg, C., Wilcke, W., & Oelmann, Y. (2015). Plant diversity shapes microbe-rhizosphere effects on P mobilisation from organic matter in soil. *Ecology Letters*, *18*(12), 1356–1365. https://doi.org/10.1111/ele.12530

Hinderling, J. & Ralph Bolliger. (2023). *Vegetation records for grassland EPs, 2008—2021* [Dataset]. Biodiversity Exploratories Information System. https://www.bexis.uni-jena.de/ddm/data/Showdata/31175?version=13

Isbell, F., Craven, D., Connolly, J., Loreau, M., Schmid, B., Beierkuhnlein, C., Bezemer, T. M., Bonin, C., Bruelheide, H., de Luca, E., Ebeling, A., Griffin, J. N., Guo, Q., Hautier, Y., Hector, A., Jentsch, A., Kreyling, J., Lanta, V., Manning, P., … Eisenhauer, N. (2015). Biodiversity increases the resistance of ecosystem productivity to climate extremes. *Nature*, *526*(7574), 574–577. https://doi.org/10.1038/nature15374

Kandeler, E., Marhan, S., Berner, D., & Boeddinghaus, R. (2021). *Microbial soil properties of all grassland EPs, soil sampling campaign (SSC) 2014, SCALEMIC* [Dataset]. Biodiversity Exploratories Information System. https://www.bexis.uni-jena.de/ddm/data/Showdata/20251?version=3

Lama, S., Velescu, A., Leimer, S., Weigelt, A., Chen, H., Eisenhauer, N., Scheu, S., Oelmann, Y., & Wilcke, W. (2020). Plant diversity influenced gross nitrogen mineralization, microbial ammonium consumption and gross inorganic N immobilization in a grassland experiment. *Oecologia*, *193*(3), 731–748. https://doi.org/10.1007/s00442-020-04717-6

Martin-Guay, M.-O., Paquette, A., Reich, P. B., & Messier, C. (2020). Implications of contrasted above- and below-ground biomass responses in a diversity experiment with trees. *Journal of Ecology*, *108*(2), 405–414. https://doi.org/10.1111/1365-2745.13265

National Ecological Observatory Network (NEON). (2024a). *Herbaceous clip harvest (DP1.10023.001)* [Dataset]. National Ecological Observatory Network (NEON). https://doi.org/10.48443/D6A4-0X67

National Ecological Observatory Network (NEON). (2024b). *Root biomass and chemistry, periodic (DP1.10067.001)* [Dataset]. National Ecological Observatory Network (NEON). https://doi.org/10.48443/NXVQ-GA39

National Ecological Observatory Network (NEON). (2024c). *Soil microbe biomass (DP1.10104.001)* [Dataset]. National Ecological Observatory Network (NEON). https://doi.org/10.48443/DBRV-4105

National Ecological Observatory Network (NEON). (2024d). *Soil physical and chemical properties, periodic (DP1.10086.001)* [Dataset]. National Ecological Observatory Network (NEON). https://doi.org/10.48443/GTMR-RP77

Ratcliffe, S., Wirth, C., Jucker, T., van der Plas, F., Scherer-Lorenzen, M., Verheyen, K., Allan, E., Benavides, R., Bruelheide, H., Ohse, B., Paquette, A., Ampoorter, E., Bastias, C. C., Bauhus, J., Bonal, D., Bouriaud, O., Bussotti, F., Carnol, M., Castagneyrol, B., … Baeten, L. (2017). Biodiversity and ecosystem functioning relations in European forests depend on environmental context. *Ecology Letters*, *20*(11), 1414–1426. https://doi.org/10.1111/ele.12849

Reich, P. (2021). *Root biomass data: BioCON : Biodiversity, Elevated CO2, and N Enrichment* [Dataset]. Environmental Data Initiative. https://doi.org/10.6073/PASTA/C3EAF35E30FC5CCBEC4CAE48192EA7B3

Reich, P. (2024a). *Nitrogen mineralization rate: BioCON : Biodiversity, Elevated CO2, and N Enrichment* [Dataset]. Environmental Data Initiative. https://doi.org/10.6073/PASTA/A7BC3CB9D2B13DE2C776B6DEA7E0EF1D

Reich, P. (2024b). *Plant aboveground biomass data: BioCON : Biodiversity, Elevated CO2, and N Enrichment* [Dataset]. Environmental Data Initiative. https://doi.org/10.6073/PASTA/C153C4EB2414BB1EC2AB8B07ADC17EF2

Rueß, L. (2021). *Forest soil PLFA concentrations (soil sampling campaign 2014)* [Dataset]. Biodiversity Exploratories Information System. https://www.bexis.uni-jena.de/ddm/data/Showdata/20926?version=3

Schall, P., Ammer, C., & Schulze, E.-D. (2017). *1st forest inventory on all forest EPs, single tree data, 2008—2014* [Dataset]. Biodiversity Exploratories Information System. https://www.bexis.uni-jena.de/ddm/data/Showdata/18268?version=2

Schenk, N. V., Penone, C., Allan, E., & Fischer, M. (2023). *Assembled ecosystem measures from grassland EPs (2008-2018) for multifunctionality synthesis—June 2020 (ID 27087)* [Dataset]. Biodiversity Exploratories Information System. https://www.bexis.uni-jena.de

Schuldt, A., Assmann, T., Brezzi, M., Buscot, F., Eichenberg, D., Gutknecht, J., Härdtle, W., He, J. S., Klein, A. M., Kühn, P., Liu, X., Ma, K., Niklaus, P. A., Pietsch, K. A., Purahong, W., Scherer-Lorenzen, M., Schmid, B., Scholten, T., Staab, M., … Bruelheide, H. (2018). Biodiversity across trophic levels drives multifunctionality in highly diverse forests. *Nature Communications*, *9*(1), 2989. https://doi.org/10.1038/s41467-018-05421-z

Solly, E. F., Schöning, I., Boch, S., Kandeler, E., Marhan, S., Michalzik, B., Müller, J., Zscheischler, J., Trumbore, S. E., & Schrumpf, M. (2014). Factors controlling decomposition rates of fine root litter in temperate forests and grasslands. *Plant and Soil*, *382*(1), 203–218. https://doi.org/10.1007/s11104-014-2151-4

Solly, E., Schöning, I., Klötzing, T., & Schrumpf, M. (2023). *Soil sampling campaign 2011, all experimental plots (EP), 0-10 cm—Root biomass* [Dataset]. Biodiversity Exploratories Information System. https://www.bexis.uni-jena.de/ddm/data/Showdata/14448?version=4

Tilman, D. (2018). *Soil microbial functions and enzyme activity:BAC: Biodiversity and Climate* [Dataset]. Environmental Data Initiative. https://doi.org/10.6073/PASTA/119D926E639CCA06B93B5F1EC6C040F1

Tilman, D. (2024a). *Plant aboveground biomass data: Biodiversity II: Effects of Plant Biodiversity on Population and Ecosystem Processes* [Dataset]. Environmental Data Initiative. https://doi.org/10.6073/PASTA/1F10CB47B9E121F3EA0361EEB1CC7BE6

Tilman, D. (2024b). *Root biomass data: Biodiversity II: Effects of Plant Biodiversity on Population and Ecosystem Processes* [Dataset]. Environmental Data Initiative. https://doi.org/10.6073/PASTA/201DE89DBF7B334B670D633D9F716CC6

van der Plas, F., Manning, P., Allan, E., Scherer-Lorenzen, M., Verheyen, K., Wirth, C., Zavala, M. A., Hector, A., Ampoorter, E., Baeten, L., Barbaro, L., Bauhus, J., Benavides, R., Benneter, A., Berthold, F., Bonal, D., Bouriaud, O., Bruelheide, H., Bussotti, F., …

Fischer, M. (2016). Jack-of-all-trades effects drive biodiversity–ecosystem multifunctionality relationships in European forests. *Nature Communications*, *7*, 11109.

van der Plas, F., Schröder-Georgi, T., Weigelt, A., Barry, K., Meyer, S., Alzate, A., Barnard, R. L., Buchmann, N., de Kroon, H., Ebeling, A., Eisenhauer, N., Engels, C., Fischer, M., Gleixner, G., Hildebrandt, A., Koller-France, E., Leimer, S., Milcu, A., Mommer, L., … Wirth, C. (2020). Plant traits alone are poor predictors of ecosystem properties and long-term ecosystem functioning. *Nature Ecology & Evolution*, *4*(12), 1602–1611. https://doi.org/10.1038/s41559-020-01316-9
